# Author Correction: An extra-erythrocyte role of haemoglobin body in chondrocyte hypoxia adaption

**DOI:** 10.1038/s41586-024-08057-w

**Published:** 2024-09-25

**Authors:** Feng Zhang, Bo Zhang, Yuying Wang, Runmin Jiang, Jin Liu, Yuexian Wei, Xinyue Gao, Yichao Zhu, Xinli Wang, Mao Sun, Junjun Kang, Yingying Liu, Guoxing You, Ding Wei, Jiajia Xin, Junxiang Bao, Meiqing Wang, Yu Gu, Zhe Wang, Jing Ye, Shuangping Guo, Hongyan Huang, Qiang Sun

**Affiliations:** 1 Department of Pathology, School of Basic Medicine and Xijing Hospital, State Key Laboratory of Cancer Biology, Air Force Medical Center, The Fourth Military Medical University, Xi’an, China; 2 Frontier Biotechnology Laboratory, Beijing Institute of Biotechnology, Academy of Military Medical Science; Research Unit of Cell Death Mechanism, 2021RU008, Chinese Academy of Medical Science, Beijing, China; 3 Department of Oncology, Beijing Shijitan Hospital of Capital Medical University, Beijing, China; 4 Nanhu Laboratory, Jiaxing, China; 5 Department of Thoracic Surgery, Tangdu Hospital, The Fourth Military Medical University, Xi’an, China; 6 Department of Orthopedics, Xijing Hospital, The Fourth Military Medical University, Xi’an, China; 7 Department of Biochemistry and Molecular Biology, The Fourth Military Medical University, Xi’an, China; 8 Department of Neurobiology, The Fourth Military Medical University, Xi’an, China; 9 Institute of Health Service and Transfusion Medicine, Academy of Military Medical Sciences, Beijing, China; 10 Department of Cell Biology, National Translational Science Center for Molecular Medicine, State Key Laboratory of Cancer Biology, The Fourth Military Medical University, Xi’an, China; 11 Department of Blood Transfusion, Xijing Hospital, The Fourth Military Medical University, Xi’an, China; 12 Department of Aerospace Hygiene, The Fourth Military Medical University, Xi’an, China; 13 Department of Oral Anatomy and Physiology, School of Stomatology, The Fourth Military Medical University, Xi’an, China

**Keywords:** Cartilage development, Mechanisms of disease, Cell death

Correction to: *Nature* 10.1038/s41586-023-06611-6 Published online 4 October 2023

In the version of the article initially published, the TUNEL images in the lower panels of Supplementary Fig. 9f and 9g were incorrect and have now been corrected in the online version of the article.

