# Supplementary material for: An extra-erythrocyte role of haemoglobin body in chondrocyte hypoxia adaption
Source: Nature. 2023 Oct 4;622(7984):834–41. doi: 10.1038/s41586-023-06611-6 (PMC10600011; doi:10.1038/s41586-023-06611-6)
Supplement: Supplementary file 1 — This file contains Supplementary Figures 1–14. [file 41586_2023_6611_MOESM1_ESM.pdf]

nature portfolio

https://doi.org/10.1038/s41586-023-06611-6

Supplementary information

An extra-erythrocyte role of haemoglobin body in chondrocyte hypoxia adaption

In the format provided by the authors and unedited

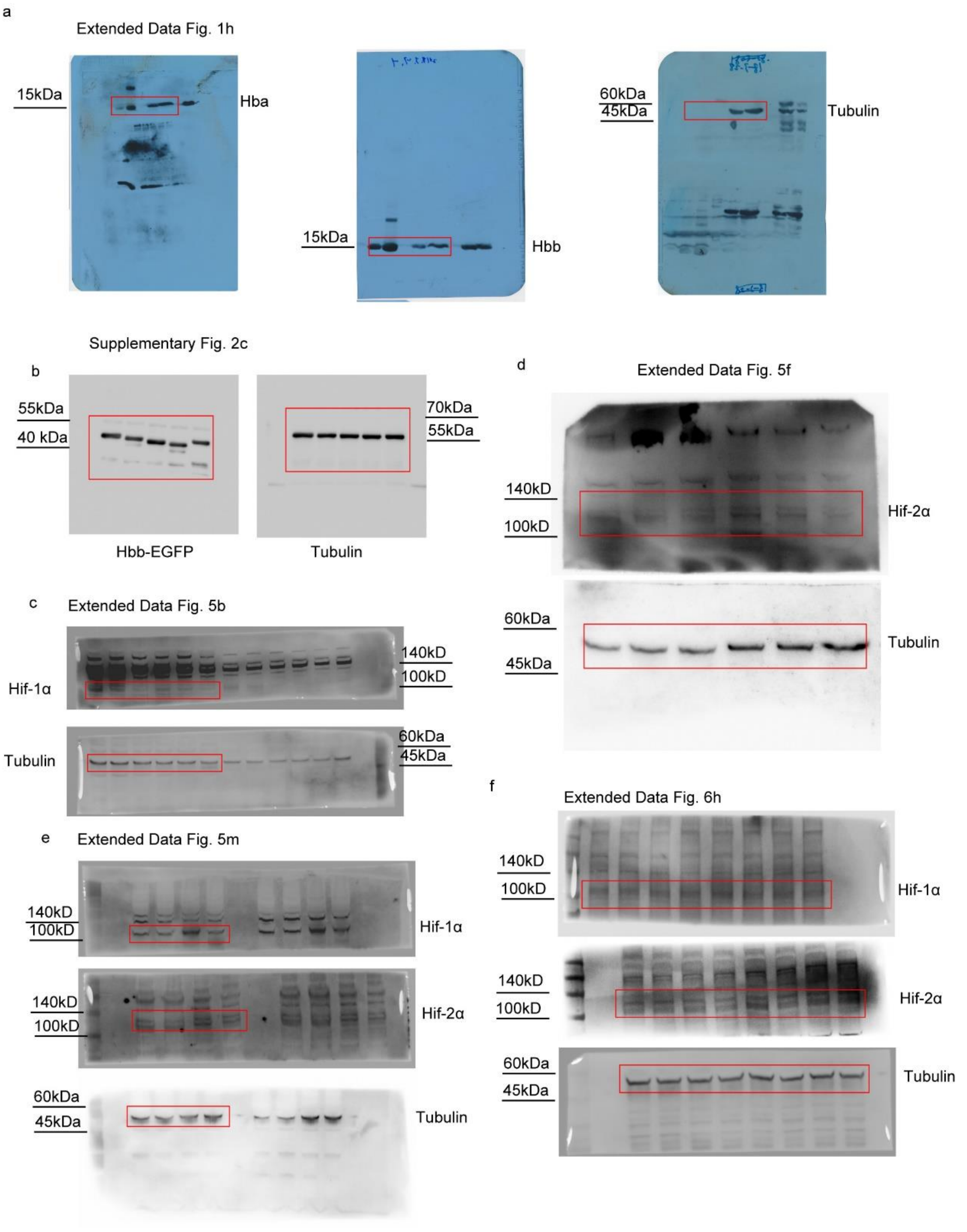

Supplementary Fig. 1 (a-f) ｜Original source images for Western blot data. Original Western blot images of Extended Data and Supplementary Figures. All images of replicates per experiment or repeated experiments were shown.

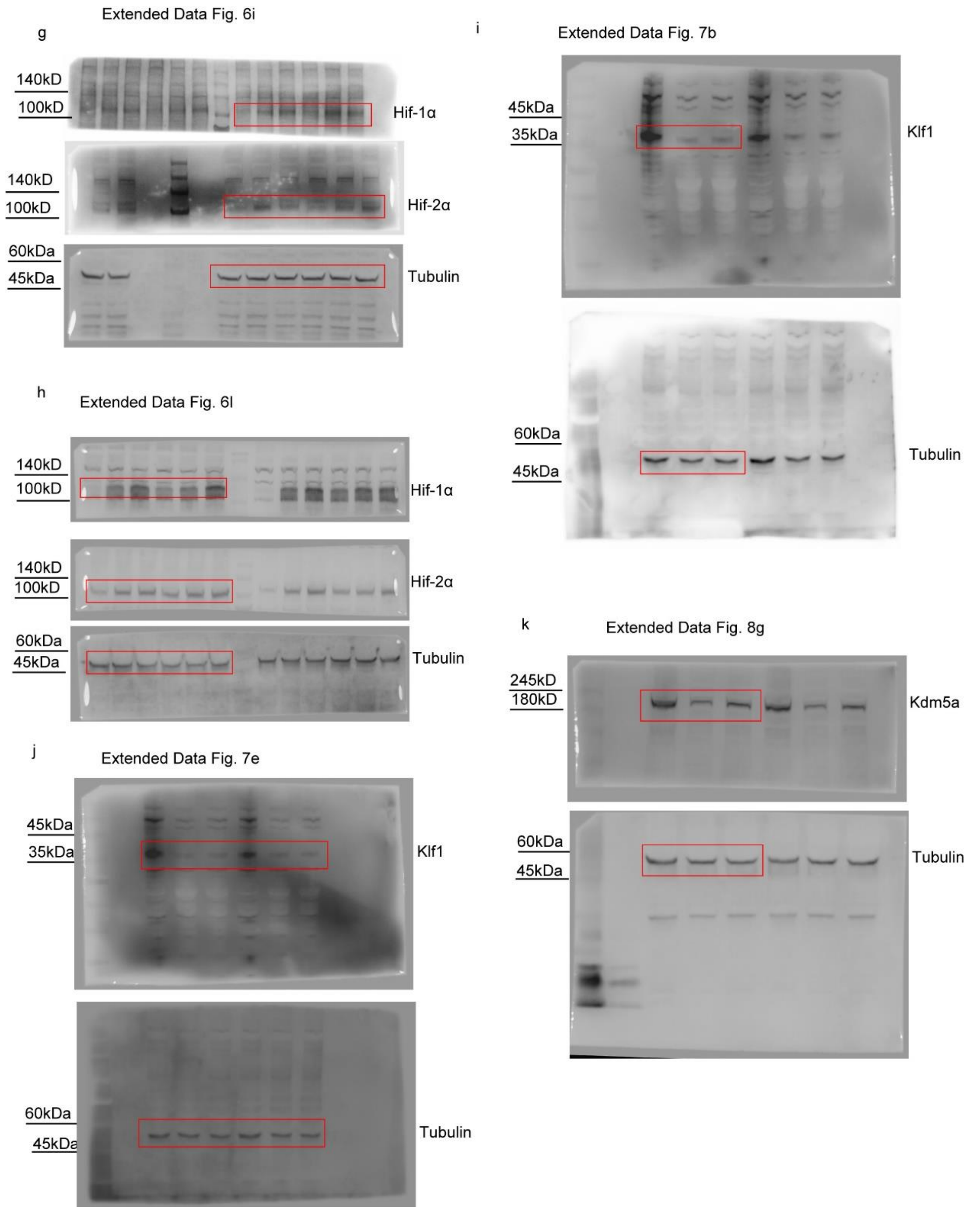

Supplementary Fig. 1 (g-k) ｜Original source images for Western blot data. Original Western blot images of Extended Data and Supplementary Figures. All images of replicates per experiment or repeated experiments were shown.

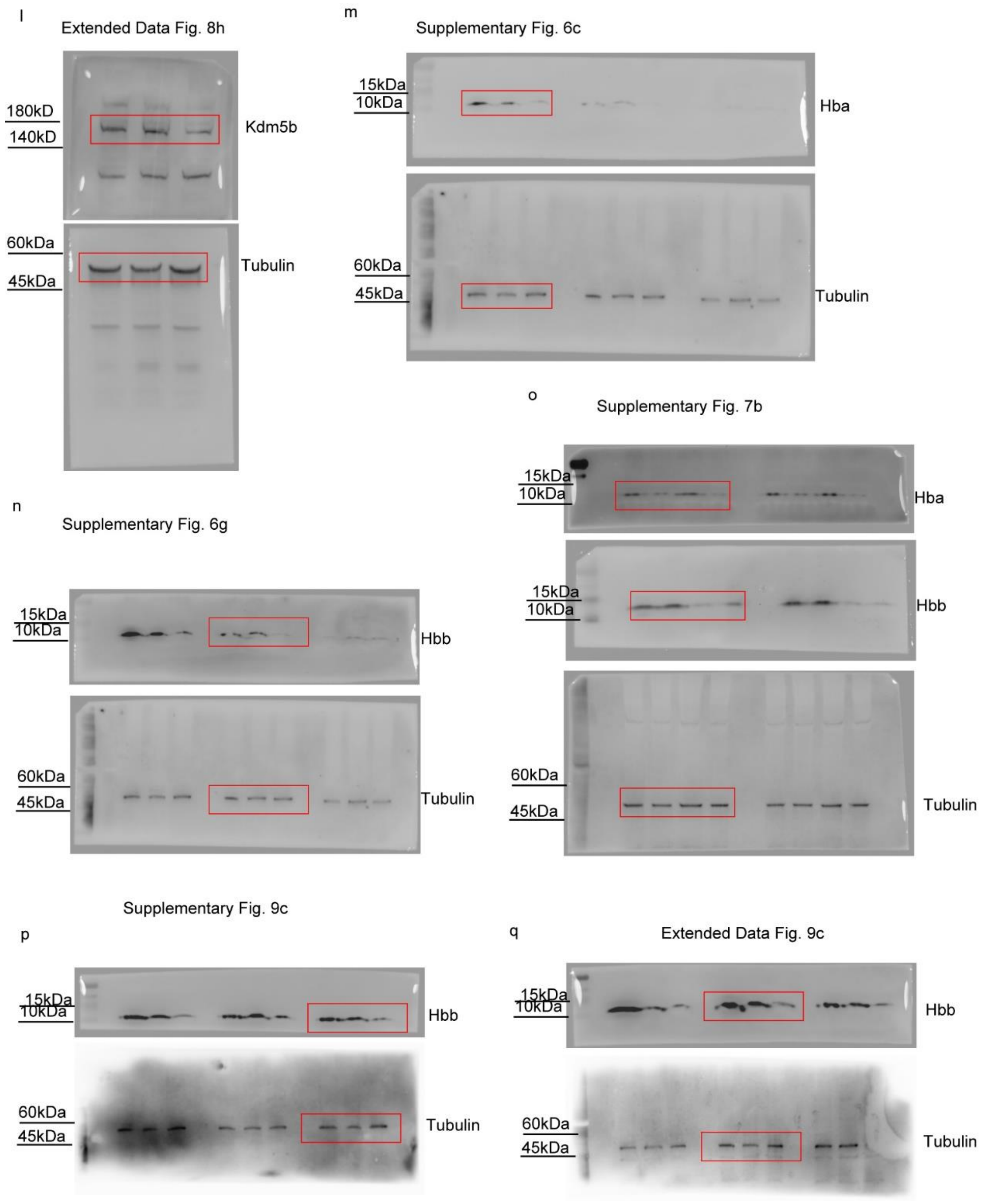

Supplementary Fig. 1 (i-q) ｜Original source images for Western blot data. Original Western blot images of Extended Data and Supplementary Figures. All images of replicates per experiment or repeated experiments were shown.

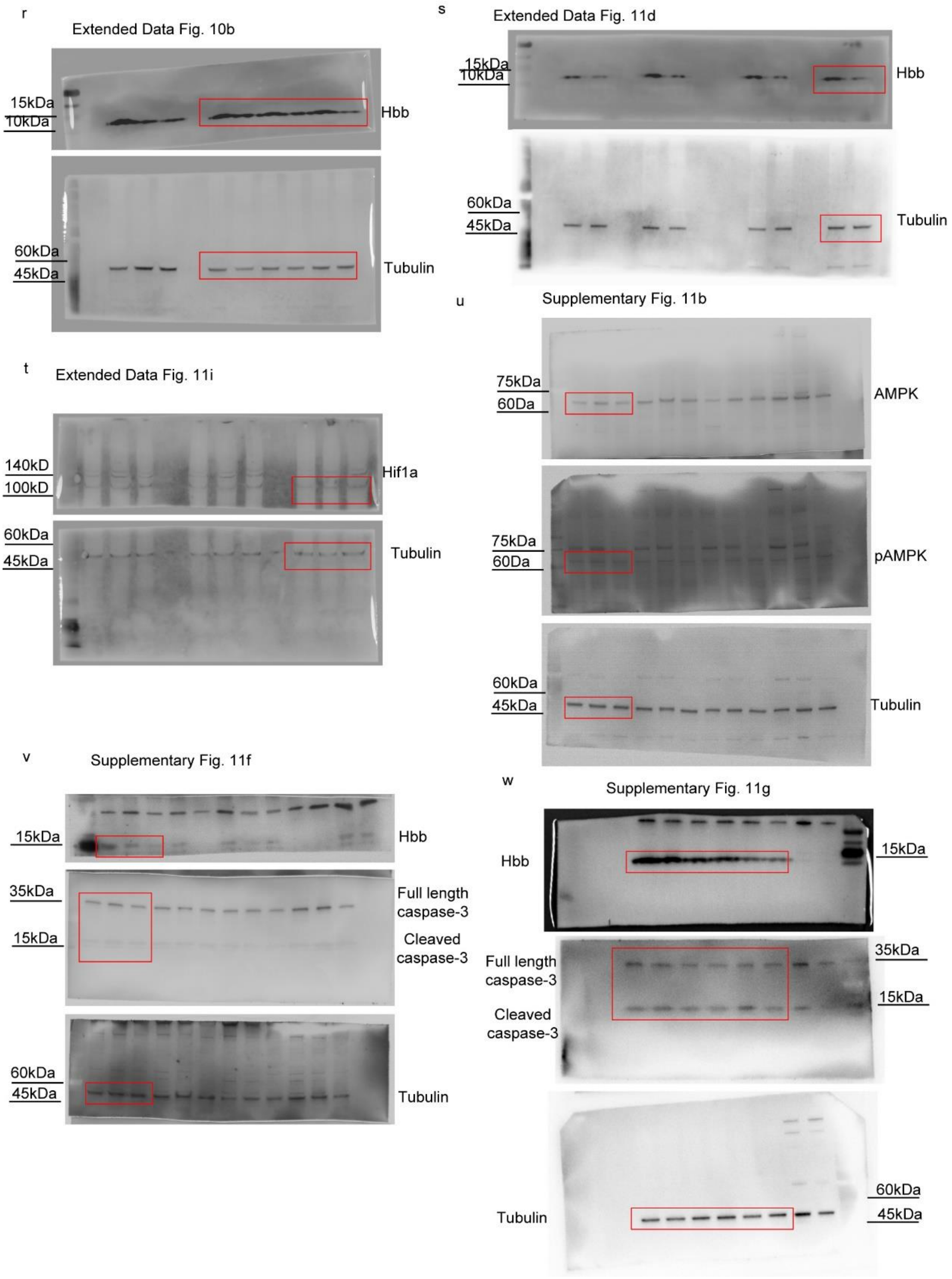

Supplementary Fig. 1 (r-w) | Original source images for Western blot data. Original Western blot images of Extended Data and Supplementary Figures. All images of replicates per experiment or repeated experiments were shown.

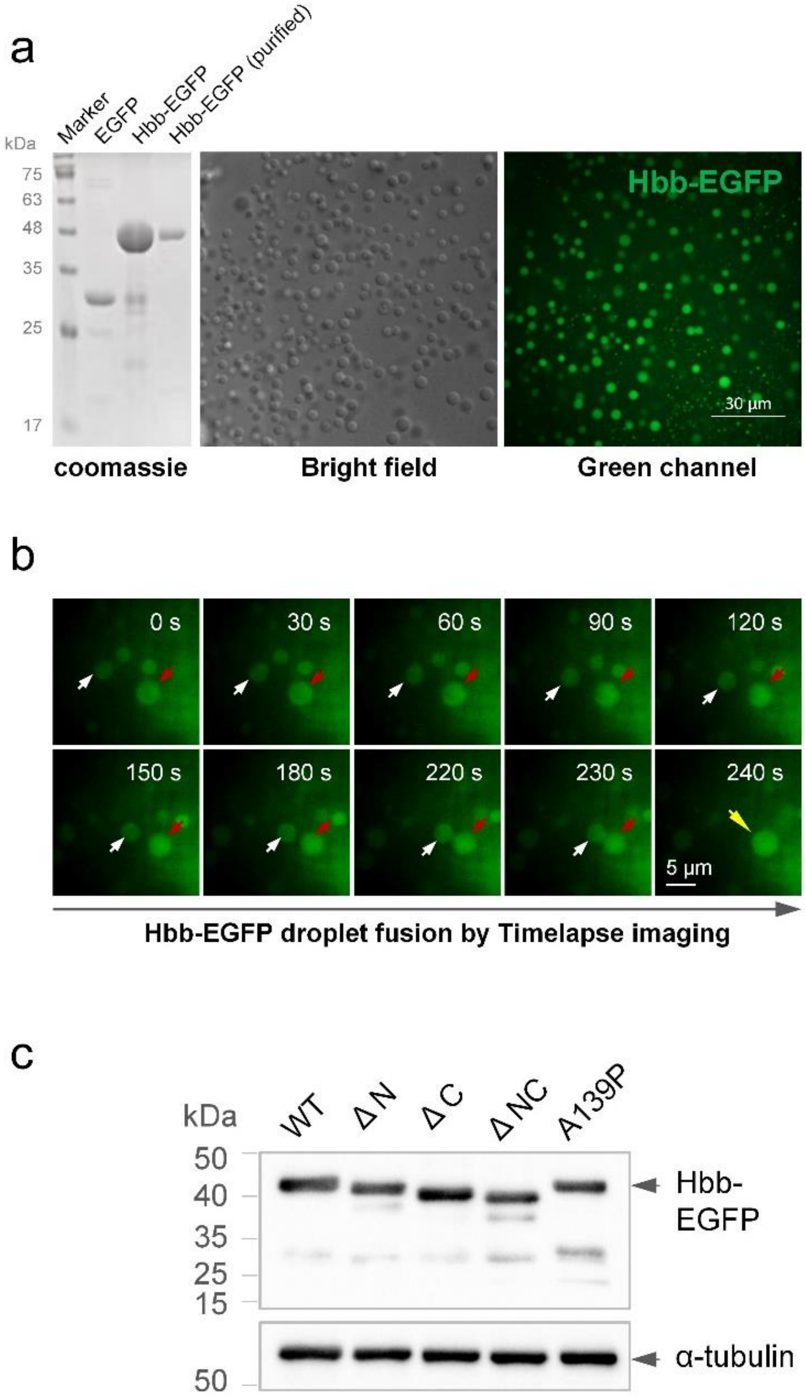

Supplementary Fig. 2 | Liquid phase separation of Hbb-EGFP *in vitro*. a, Coomassie brilliant blue stained gel of the purified Hbb-EGFP protein (left panel) and liquid phase separation of Hbb-EGFP in LLPS buffer (150 mM KH_2PO_4/K_2HPO_4, pH 7.35, PEG2000 10% (w/v)). **b**, Timelapse imaging of Hbb-EGFP droplet fusion, white arrows and red arrows indicate droplets before fusion, yellow arrow indicates fused droplet. Scale bar: 5 µm. **c,** Western blot analysis of Hbb-EGFP and its mutant expression in 293T cells. For gel source data, see Supplementary Fig.1b.

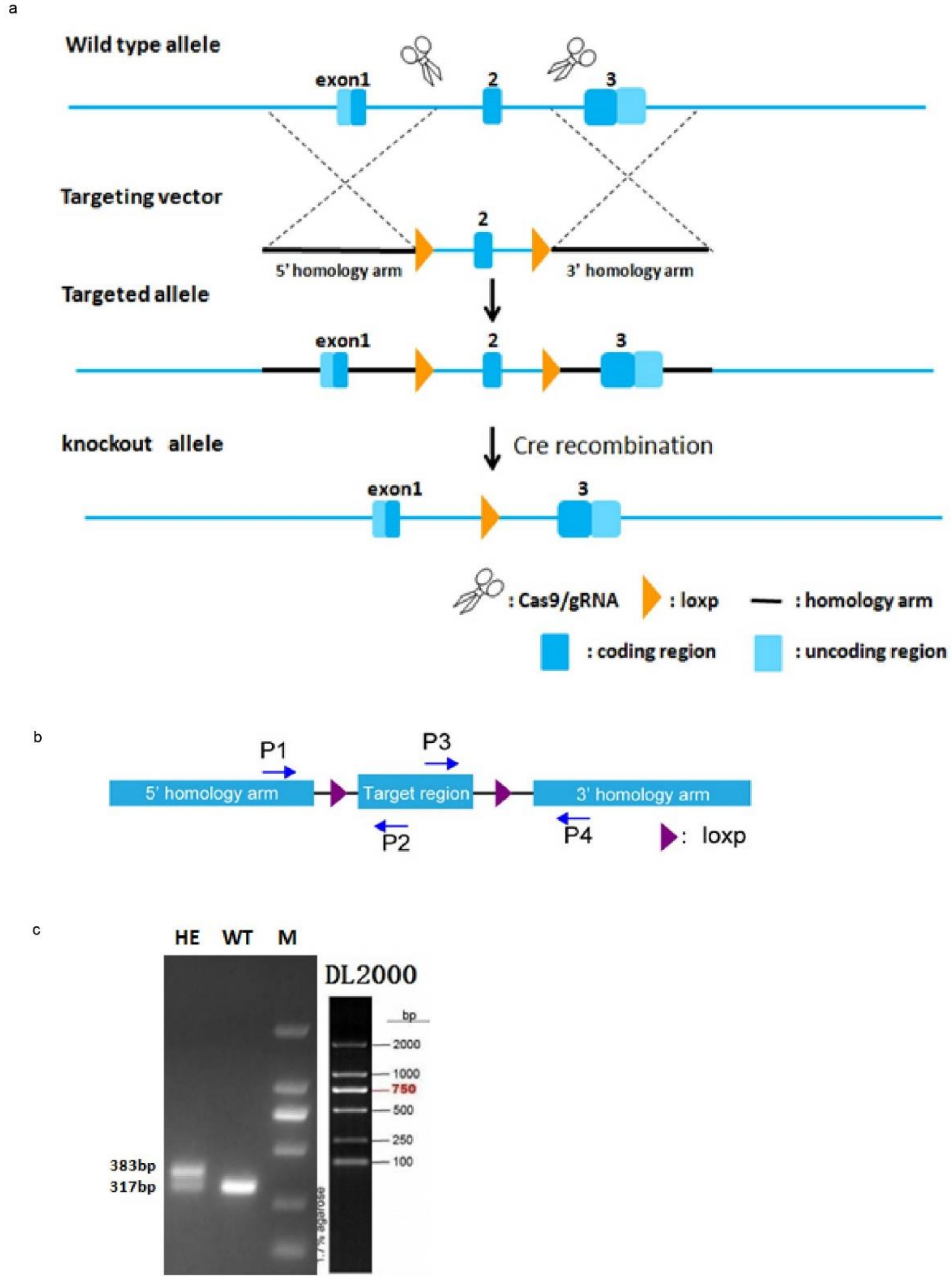

Supplementary Fig. 3 | Generation of *Klf1*$^{F/F}$ mice.a, A targeting vector was designed to replace the exon 2 of *Klf1* (*Klf1*$^{F/F}$). **b, c**, Genotyping by amplifying the specific WT (317 bp) and mutant (383 bp) DNA fragments by PCR with the P1 and P2 primer pair.

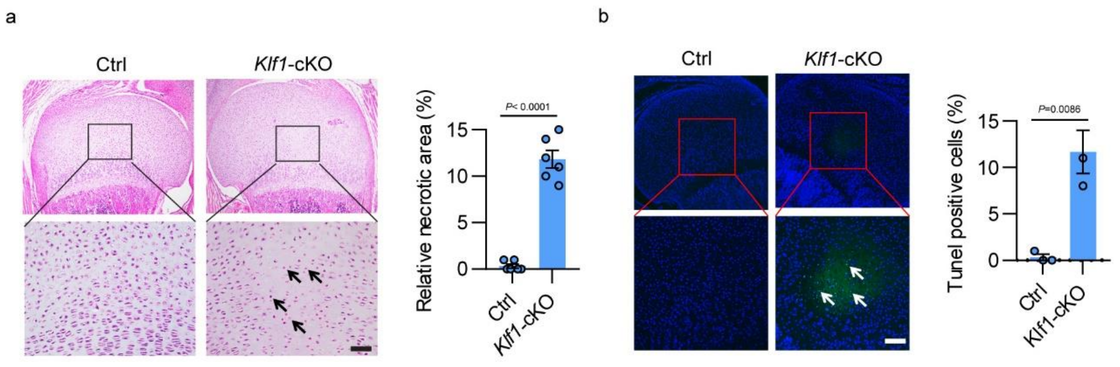

Supplementary Fig. 4 | Phenotypes of *Klf1* conditional knockout mice. a, Conditional knockout of *Klf1* resulted in massive cell death in the center of cartilaginous growth plate. Quantification of all biological replicates is provided on the right. Scale bar: 50 µm. n=6. Error bars represent SEM. P values were calculated using two-tailed Student's t-test. **b**, TUNEL assay of the proximal humeral cartilages of *Klf1*$^{F/F}$ (Ctrl) or *Klf1*$^{F/F}$/*Col2a1*- *Cre*^{ERT2} (*Klf1*-cKO) growth plates of P5 mice, which were treated by tamoxifen (100mg/kg) for 4 days. Quantification of all biological replicates is provided on the right. Arrows indicated dead chondrocytes. Green fluorescence marked the dead cells. Scale bars, 50 µm. n = 3. Error bars represent SEM. P values were calculated using two-tailed Student's t-test. The exact P-values of comparison are presented in the figures, respectively.

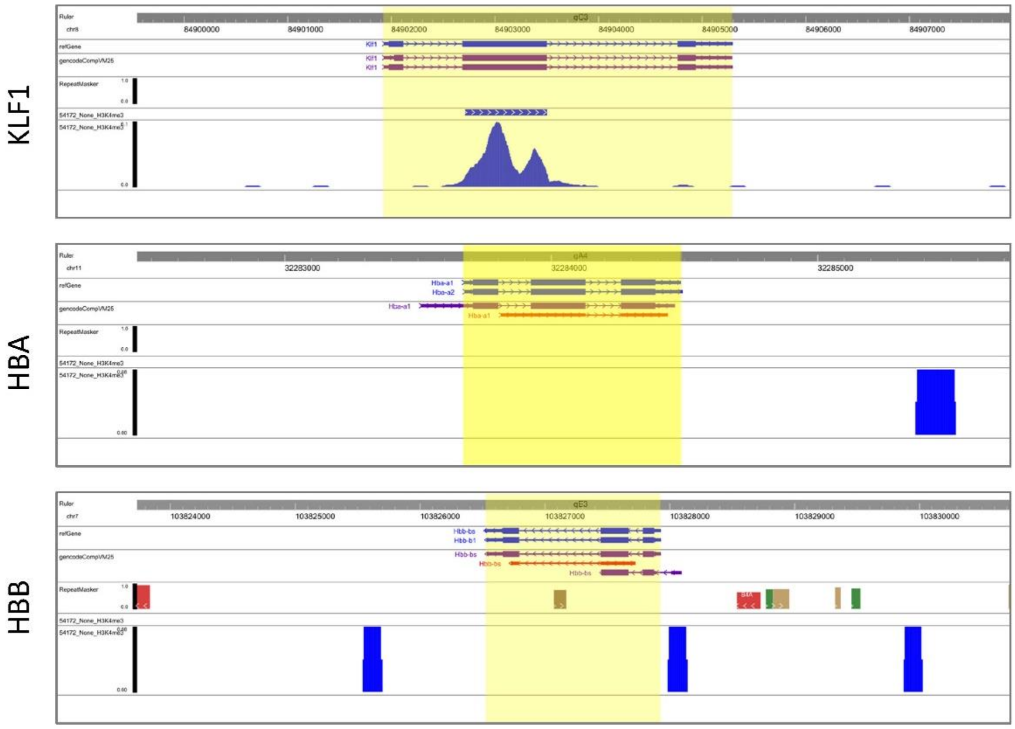

Supplementary Fig. 5 | Histone H3 Lys 4 trimethylation ($H_3K_4me_3$) level at the region of the indicated genes. Results were obtained at website: http://cistrome.org/db/#/, source data were derived from ChIP-seq data provided by Ohba S, et al (Cell Rep, 2015).

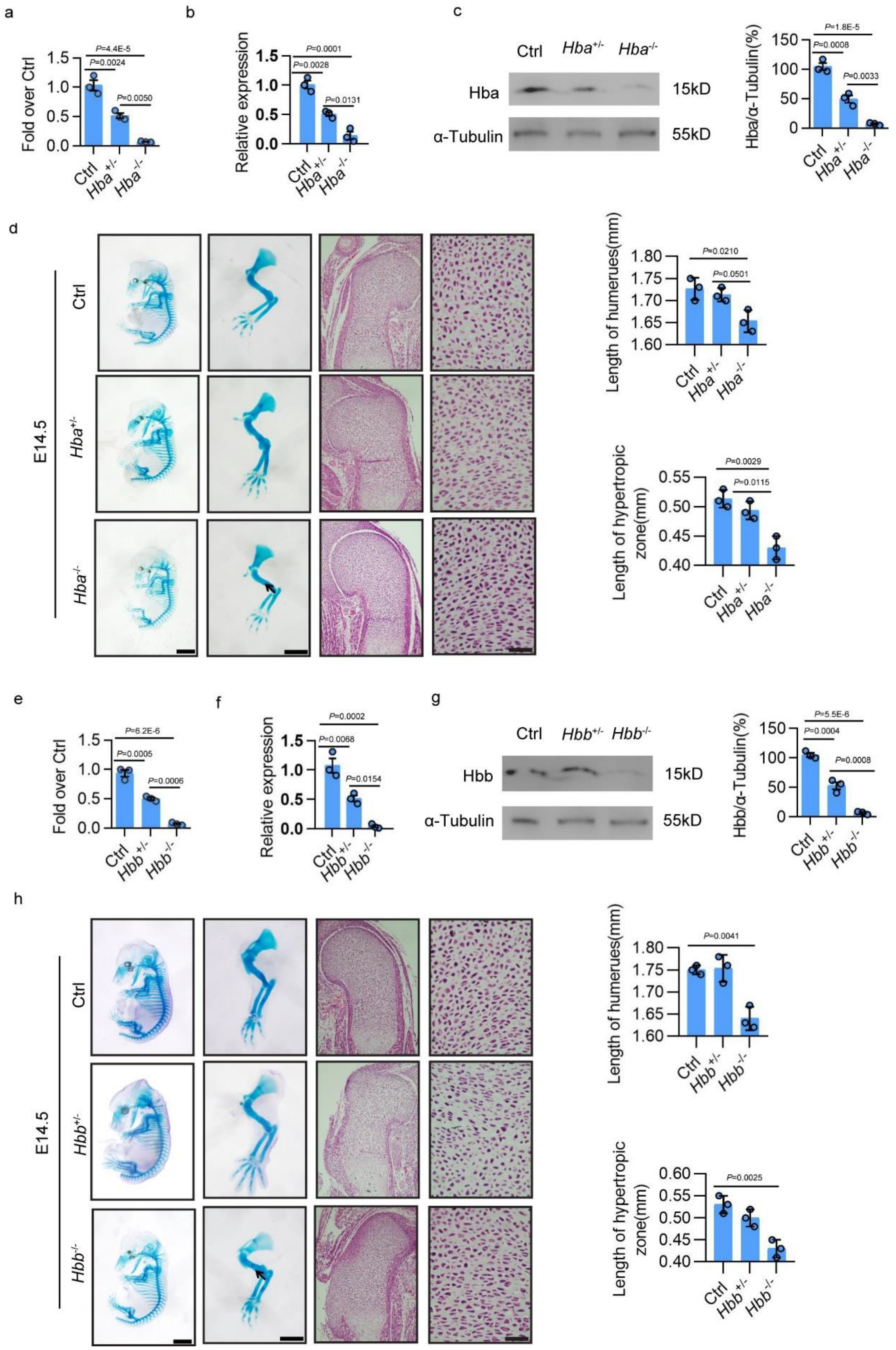

Supplementary Fig. 6 | Skeleton analysis of hemoglobin knockout mice at E14.5. a, qPCR of genomic DNA extracted from *Hba*$^{+/+}$(Ctrl), *Hba*$^{+/-}$ or *Hba*$^{-/-}$ growth plates of E14.5 mice. Data were normalized to β2-microglobulin (n=3 biologically independent samples). **b,** Quantification of

Hba mRNA by qRT-PCR of total RNA extracted from E14.5 $Hba^{+/+}$(Ctrl), $Hba^{+/-}$ or $Hba^{-/-}$ growth plates. Data were normalized to *Gapdh* (n=3 biologically independent samples). **c,** Quantification of Hba protein by Western blot analysis of total protein lysate extracted from E14.5 $Hba^{+/+}$(Ctrl), $Hba^{+/-}$ or $Hba^{-/-}$ growth plates. A representative Western blot is shown on the left, and quantification of all biological replicates is provided on the right. Data were normalized to α-tubulin (n=3 biologically independent samples). For gel source data, see Supplementary Fig.1m. **d.** Skeleton analysis indicated mild delay of cartilage hypertrophy in mice of homozygous knockout of *Hba*. Black arrow indicated the delayed hypertrophic regions. Scale bars: 1.5 mm (left), 0.5 mm (right). n=3 biologically independent samples. No noticeable pattern defect of cartilaginous growth plates of E14.5 mice was detected upon knockout of *Hba* by histology examination. Scale bar, 50 μm. n=3 biologically independent samples. For gel source data, see Supplementary Fig.1m. **e,** qPCR of genomic DNA extracted from $Hbb^{+/+}$(Ctrl), $Hbb^{+/-}$ or $Hbb^{-/-}$ growth plates of E14.5 mice. Data were normalized to β2-microglobulin (n = 3). **f,** Quantification of *Hbb* mRNA by qRT-PCR of total RNA extracted from E14.5 $Hbb^{+/+}$(Ctrl), $Hbb^{+/-}$ or $Hbb^{-/-}$ growth plates. Data were normalized to *Gapdh* (n=3 biologically independent samples). **g,** Quantification of Hbb protein by Western blot analysis of total protein lysate extracted from E14.5 $Hbb^{+/+}$(Ctrl), $Hbb^{+/-}$ or $Hbb^{-/-}$ growth plates. A representative Western blot is shown on the left, and quantification of all biological replicates is provided on the right. Data were normalized to α-tubulin (n=3 biologically independent samples). For gel source data, see Supplementary Fig.1n. **h,** Skeleton analysis indicated mild delay of cartilage hypertrophy in mice of homozygous knockout of *Hbb*. Black arrow indicated the delayed hypertrophic regions. Scale bars: 1.5 mm (left), 0.5 mm (right). n=3 biologically independent samples. No noticeable pattern defect of cartilaginous growth plates of E14.5 mice was detected upon knockout of *Hbb* by histology examination. Scale bar, 50 μm. n=3 biologically independent samples. Error bars represent SEM. *P* values were calculated using one-way ANOVA tests (**a-h**). The exact *P*-values of comparison are presented in the figures, respectively.

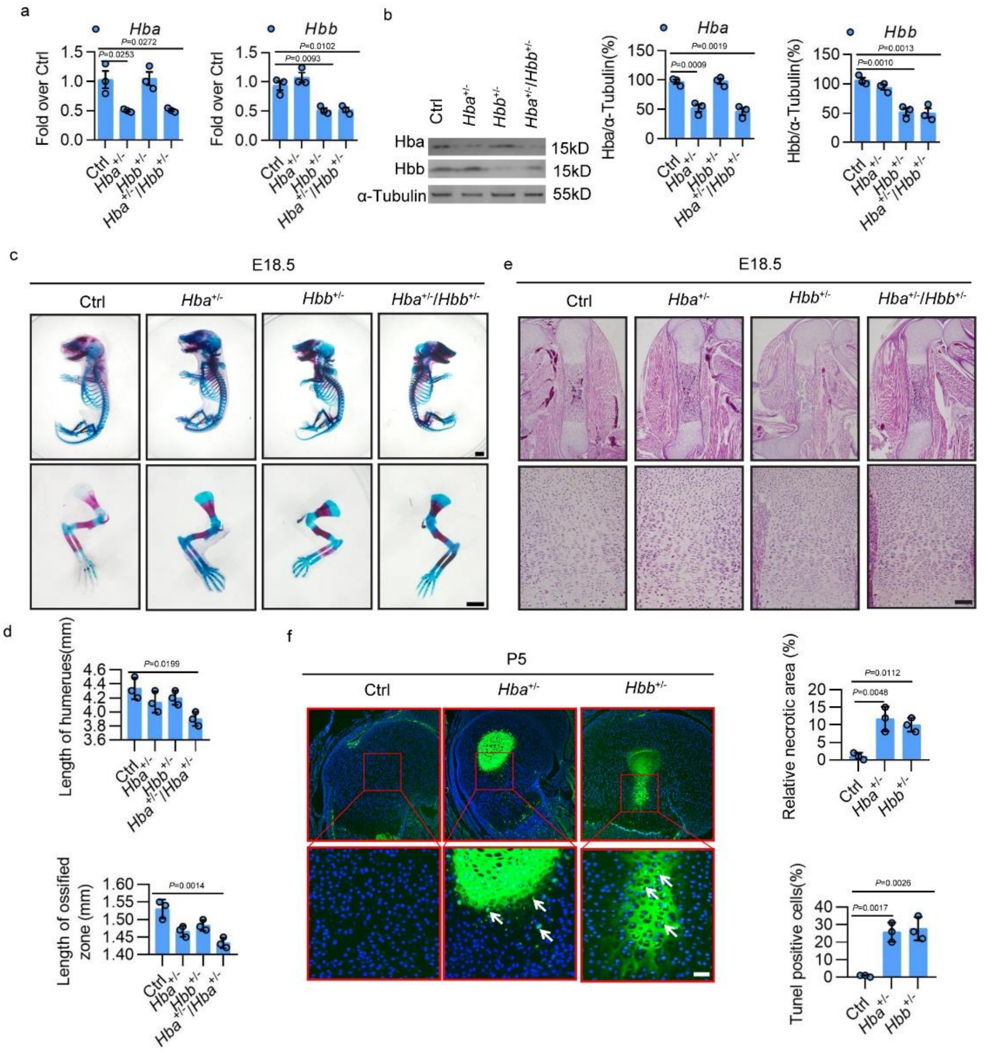

Supplementary Fig. 7 | Phenotypes of heterozygous hemoglobin knockout mice at E18.5. a, *Hba* and *Hbb* qPCR of genomic DNA extracted from wildtype (Ctrl), *Hba*$^{+/-}$, *Hbb*$^{+/-}$ or *Hba*$^{+/-}$/*Hbb*$^{+/-}$ growth plates of E18.5 mice. Data were normalized to β2-microglobulin (n=3 biologically independent samples). **b,** Quantification of Hba and Hbb protein by Western blot analysis of total protein lysate extracted from E18.5 wildtype (Ctrl), *Hba*$^{+/-}$, *Hbb*$^{+/-}$ or *Hba*$^{+/-}$/*Hbb*$^{+/-}$ growth plates. Quantification of all biological replicates is provided on the right. Data were normalized to α-tubulin (n=3 biologically independent samples). For gel source data, see Supplementary Fig.1o. **c, d,** Skeleton analysis indicated mild phenotypes in the length of humerus and ossified zone of *Hba/Hbb* double heterozygous knockout mice. Quantification of all biological replicates is provided (**d**). Scale bars: 3 mm (up), 1.5 mm (low). n=3 biologically independent samples. **e,** No obvious pattern defect of cartilaginous growth plates was detected in *Hba*/*Hbb* double heterozygous knockout mice at E18.5 by histology examination. Scale bar, 100 µm. n=6 biologically independent samples. **f,** TUNEL assay of proximal humeral cartilages of P5 mice. Quantification of all biological replicates is provided on the right. Control: wild type mice, Hba$^{+/-}$ and *Hbb*$^{+/-}$: the heterozygous littermates of *Hba* or *Hbb* genes, respectively. Arrows and green fluorescence marked the dead cells. Scale bars, 50 µm. n=3 biologically independent samples. Error bars represent SEM. *P* values were calculated using one-way ANOVA tests (**a**, **b**, **d**, **f**). The exact *P*-values of comparison are presented in the figures, respectively.

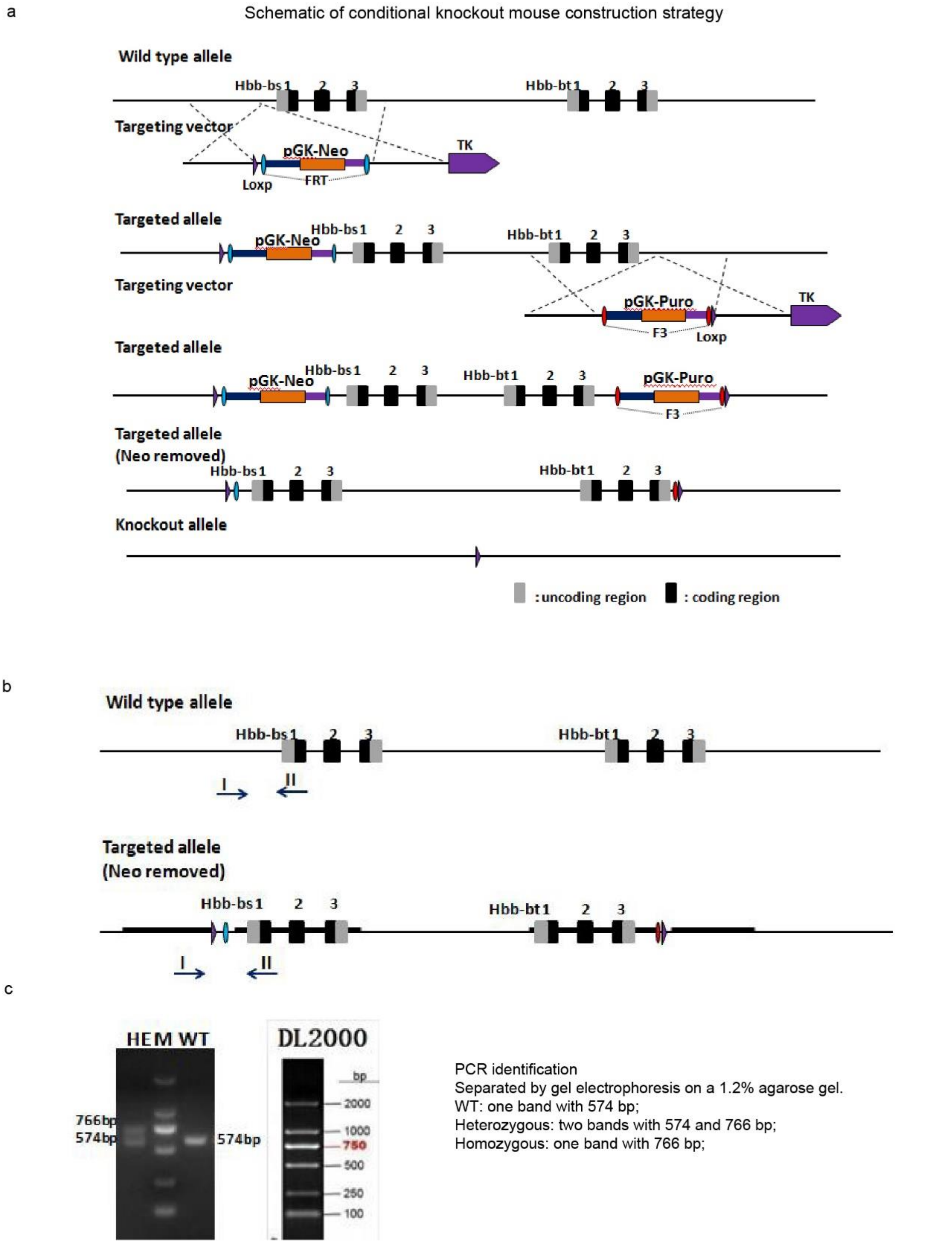

Supplementary Fig. 8 | Generation of *Hbb*$^{F/F}$ mice. a, A targeting vector was designed to replace the region spanning the 1st exon to the 3rd exon of both *Hbb-bs* and *Hbb-bt* (*Hbb*$^{F/F}$). **b, c**, Genotyping by amplifying the specific WT (574 bp) and mutant (766 bp) DNA fragments by PCR with the P1 and P2 primer pair.

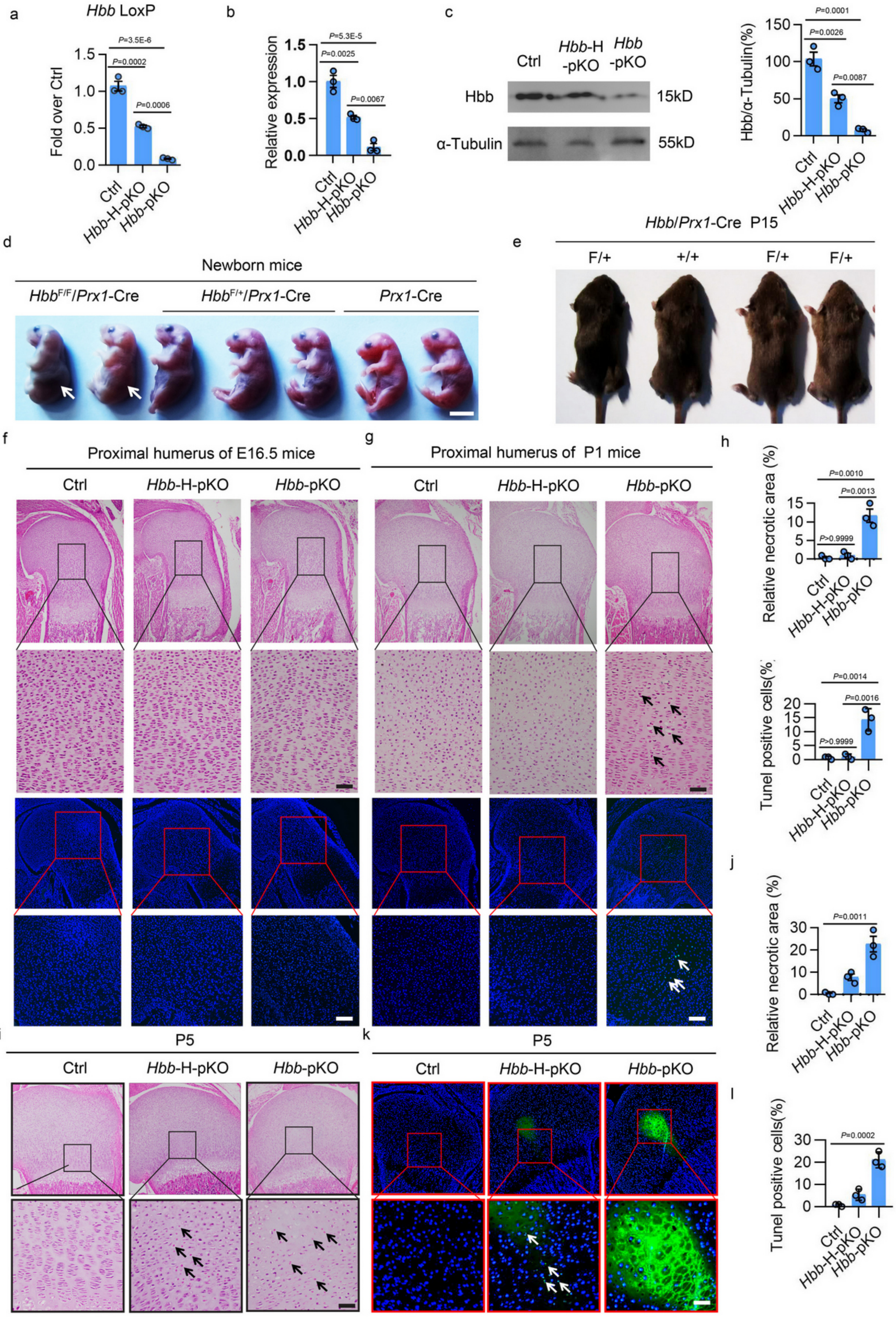

Supplementary Fig. 9 | Phenotypes of mice with *Hbb* conditional knockout by *Prx1-Cre*. a, *Hbb*-LoxP qPCR of genomic DNA extracted from *Hbb*$^{F/F}$ (Ctrl), *Hbb*$^{F/+}$/*Prx1-Cre* (heterozygous deletion, *Hbb*-H-pKO) or *Hbb*$^{F/F}$/*Prx1-Cre* (*Hbb*-pKO) growth plates of P5 mice. Data were

normalized to β2-microglobulin (n=3 biologically independent samples). **b,** Quantification of *Hbb* mRNA by qRT-PCR of total RNA extracted from P5 *Hbb*$^{F/F}$ (Ctrl), *Hbb*-H-pKO or *Hbb*-pKO growth plates. Data were normalized to *Gapdh* (n=3 biologically independent samples). **c,** Quantification of Hbb protein by Western blot analysis of total protein lysate extracted from P5 *Hbb*$^{F/F}$ (Ctrl), *Hbb*-H-pKO or *Hbb*-pKO growth plates. A representative Western blot is shown on the left, and quantification of all biological replicates is provided on the right. Data were normalized to α-tubulin (n=3 biologically independent samples). For gel source data, see Supplementary Fig.1p. **d,** Most *Hbb*$^{F/F}$/*Prx1-Cre* neonatal mice were pale and died within 1~7 days after birth. White arrows indicated dead neonatal mice. Scale bar, 3 mm. **e,** Mice with *Hbb* heterozygous deletion in mesenchymal cells survived to adult and were fertile. Scale bar, 10 mm. **f,** Histology examination (two upper panels) or TUNEL assay (two bottom panels) of proximal humeral cartilages of E16.5 mice with genotype of *Hbb*$^{F/F}$ (Ctrl), *Hbb*-H-pKO or *Hbb*-pKO. Scale bars, 50 μm. n=3 biologically independent samples. **g, h,** Histology examination (two upper panels) or TUNEL assay (two bottom panels) of *Hbb*$^{F/F}$ (Ctrl), *Hbb*-H-pKO or *Hbb*-pKO proximal humeral cartilages of P1 mice. Quantification of all biological replicates is provided (**h**). Arrows indicated dead chondrocytes. Green fluorescence marked the dead cells. Scale bars, 50 μm. n=3 biologically independent samples. **i-l** P5 littermates of *Hbb*-pKO mice exhibited massive cell death in the inner zones of developing growth plates by histology examination (**i**) or TUNEL assay (**k**). Quantification of all biological replicates is provided (**j**, **l**). Arrows indicated dead chondrocytes. Scale bar: 50 μm. n=3 biologically independent samples. Error bars represent SEM. *P* values were calculated using one-way ANOVA tests (**a-c**, **h**, **j**, **l**). The exact *P*-values of comparison are presented in the figures, respectively.

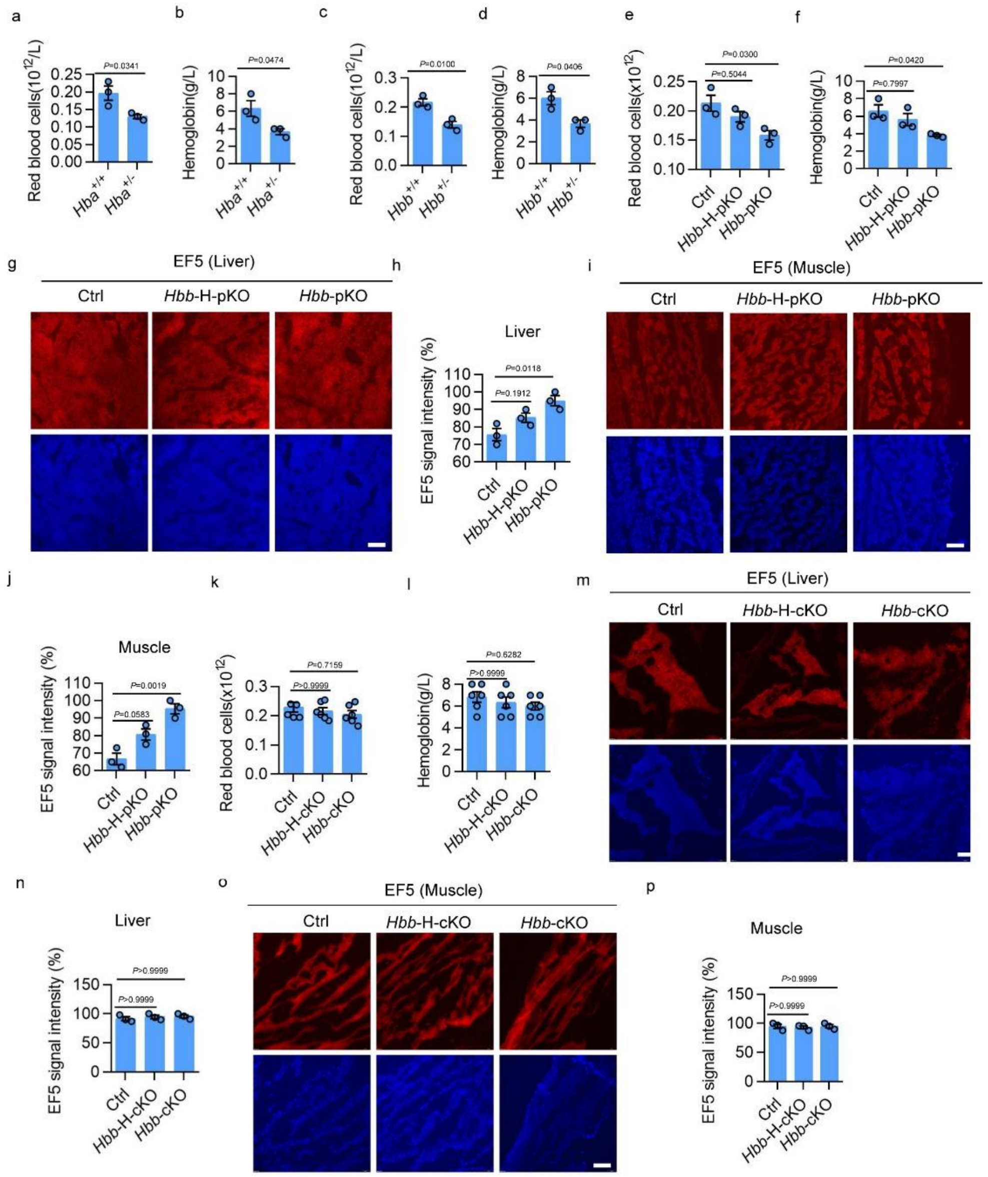

Supplementary Fig. 10 | Examination of tissue hypoxia upon hemoglobin knockout. a-f, Red blood cells counts (**a**, **c**, **e**) and hemoglobin levels (**b**, **d**, **f**) in P5 mice with indicated genotypes. Data are presented as mean ± SEM; n=3 biologically independent samples. **g-j,** Representative images and quantification of all biological replicates for EF5 staining of livers (**g**, **h**) or muscles (**i**, **j**) of *Hbb*$^{F/F}$(Ctrl), *Hbb*$^{F/+}$/*Prx1-Cre* (heterozygous deletion, *Hbb*-H-pKO) or *Hbb*$^{F/F}$/*Prx1-Cre* (*Hbb*-pKO) of P5 mice. Scale bars: 50 μm. Data are presented as mean ± SEM, Error bars represent SEM; (n=3 biologically independent samples). **k, l,** Red blood cell counts and hemoglobin levels in P5 mice with indicated genotypes. Data are presented as mean ± SEM; n=6 biologically independent samples. **m-p,** Representative images and quantification of all biological replicates for EF5 staining of livers (**m**, **n**) or muscles (**o**, **p**) of *Hbb*$^{F/F}$(Ctrl), *Hbb*$^{F/+}$/*Col2a1-Cre*^{ERT2}(heterozygous deletion, *Hbb*-H-cKO) or *Hbb*$^{F/F}$/ *Col2a1-Cre*^{ERT2} (*Hbb*-cKO) of P5 mice, which were treated by tamoxifen (100 mg/kg) for 4 days. Scale bars: 50 μm. Data are presented as mean ± SEM, Error bars represent SEM; (n=3 biologically independent samples). *P* values were calculated using two-tailed Student's *t*-test (**a**-d) or one-way ANOVA tests (**e, f, h, j-l, n, p**). The exact *P*-values of comparison are presented in the figures, respectively.

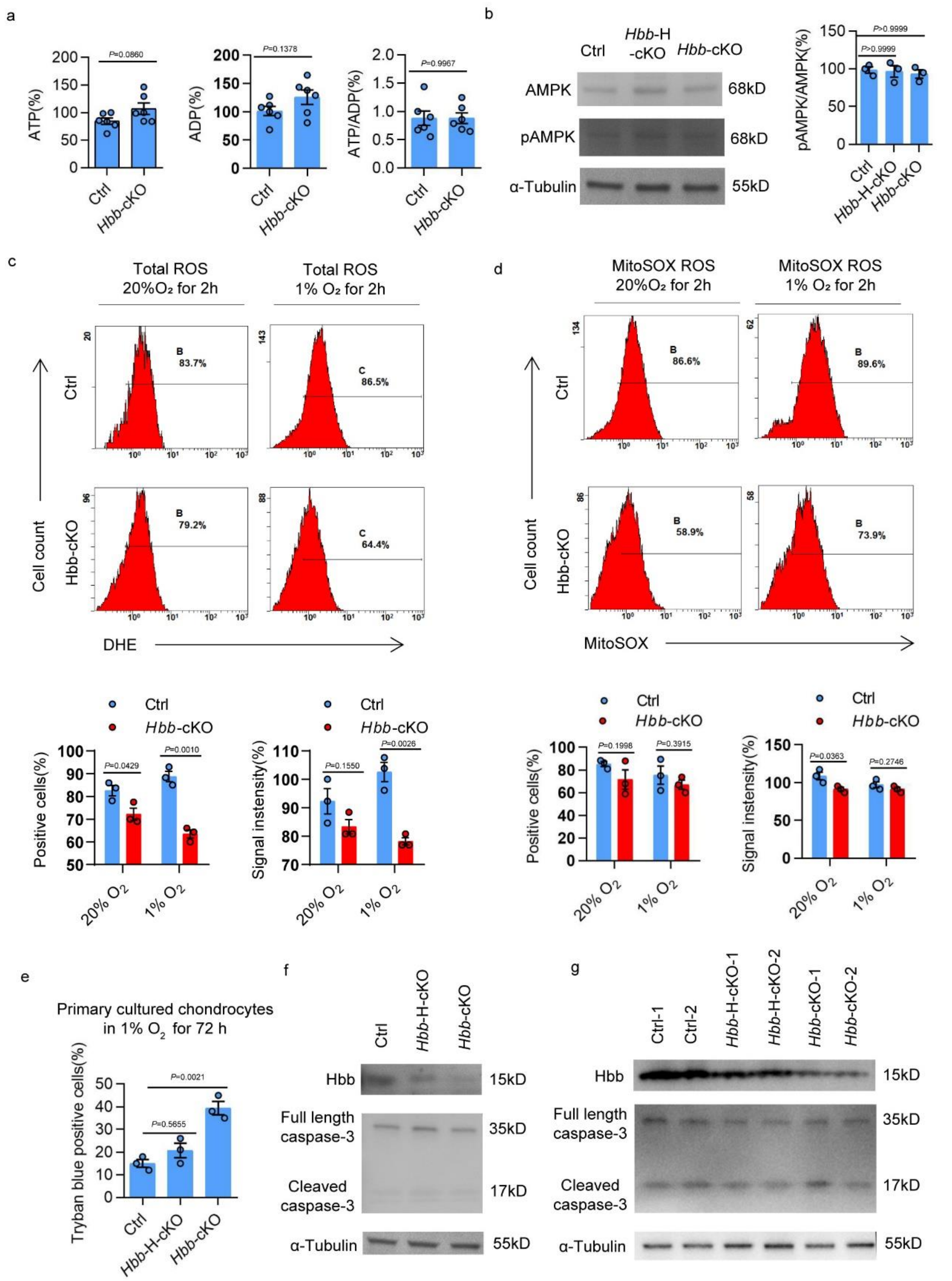

Supplementary Fig. 11 | Energy status, ROS production and apoptosis in chondrosytes with *Hbb* conditional knockout by *Col2a1-Cre*. A, Intracellular ATP, ADP, ATP/ADP ratio in chondrocytes isolated from *Hbb*$^{F/F}$(Ctrl) or *Hbb*$^{F/F}$/Col2a1^{ERT2}(Hbb-cKO) growth plates of P5 mice, which were treated by tamoxifen (100 mg/kg) for 4 days. Data were calculated as percentage of Ctrl (n=6 biologically independent samples). **B,** Quantification of AMPK and pAMPK proteins by Western blot analysis of total protein lysate extracted from chondrocytes isolated from *Hbb*$^{F/F}$ (Ctrl), *Hbb*$^{F/+}$/*Col2a1-Cre*^{ERT2} (heterozygous deletion, *Hbb*-H-cKO) or

$Hbb^{F/F}$/*Col2a1*-Cre^{ERT2} (*Hbb*-cKO) growth plates growth plates of P5 mice, which were treated by tamoxifen (100 mg/kg) for 4 days. Representative Western blot s are shown on the left and quantification of all biological replicates is provided on the right. Data for AMPK and pAMPK were normalized to α-tubulin, and calculated as percentage of the signal intensity of Ctrl (n=3 biologically independent experiments). For gel source data, see Supplementary Fig.1u. **c, d,** FACS analysis of total ROS € and mitochondrial ROS (**d**) in chondrocytes isolated from $Hbb^{F/F}$ (Ctrl) or $Hbb^{F/F}$/*Col2a1*-Cre^{ERT2} (*Hbb*-cKO) P5 growth plates, which were treated by tamoxifen (100 mg/kg) for 4 days, and cultured in 20% or 1% O_2. A representative experiment is shown at the top; quantification of all biological replicates is provided at the bottom (n=3 biologically independent samples). Signal intensity was calculated as percentage of Ctrl in 20% O_2. Data are presented as mean ± SEm. **e, f,** Counting of dead cells by trypan blue staining in primary cultured chondrocytes isolated from $Hbb^{F/F}$ (Ctrl), *Hbb*-H-cKO, and *Hbb*-cKO newborn growth plates and maintained in 1% O_2 for 72 h (n=3 biologically independent experiment). (**e**). Western blot analysis of total protein lysate extracted from primary cultured chondrocytes with an antibody specific for both full length (35 kDa) and cleaved (17–19 kDa) Caspase-3 (n=3 biologically independent experiments) (**f**). For gel source data, see Supplementary Fig.1v. **g,** Western blot analysis of total protein lysate extracted from chondrocytes of growth plates isolated from P5 $Hbb^{F/F}$ (Ctrl), $Hbb^{F/+}$/*Col2a1*-Cre^{ERT2} (heterozygous deletion, *Hbb*-H-cKO) or $Hbb^{F/F}$/ *Col2a1*-Cre^{ERT2} (*Hbb*-cKO) growth plates, which were treated by tamoxifen (100 mg/kg) for 4 days, with an antibody specific for both full length (35 kDa) and cleaved (17–19 kDa) Caspase-3. For gel source data, see Supplementary Fig.1w. n=3 biologically independent experiments. Error bars represent SEM. *P* values were calculated using two-tailed Student's *t*-test (**a**, **c**, **d**) or one-way ANOVA tests (**b**, **e**). The exact *P*-values of comparison are presented in the figures, respectively.

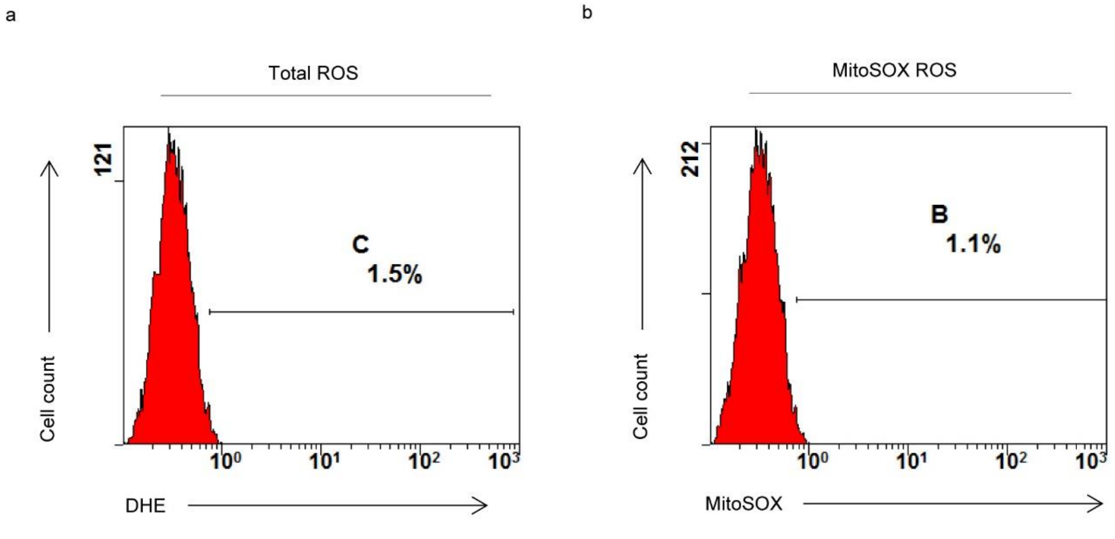

Supplementary Fig. 12｜ Gating strategies used for ROS examination by FACS. a, Gating strategy correspond to FACS data in Supplementary Fig. 11c for examination of total ROS in chondrocytes. The chondrocytes for Gating were as negative control and incubated without 5 mM dihydroethidium (DHE) probe. (n=3 biologically independent samples). **b**, Gating strategy correspond to FACS data in Supplementary Fig. 11d for examination of mitochondrial ROS in chondrocytes. The chondrocytes for Gating were as negative control and incubated without 5 mM MitoSOX probe. (n=3 biologically independent samples).

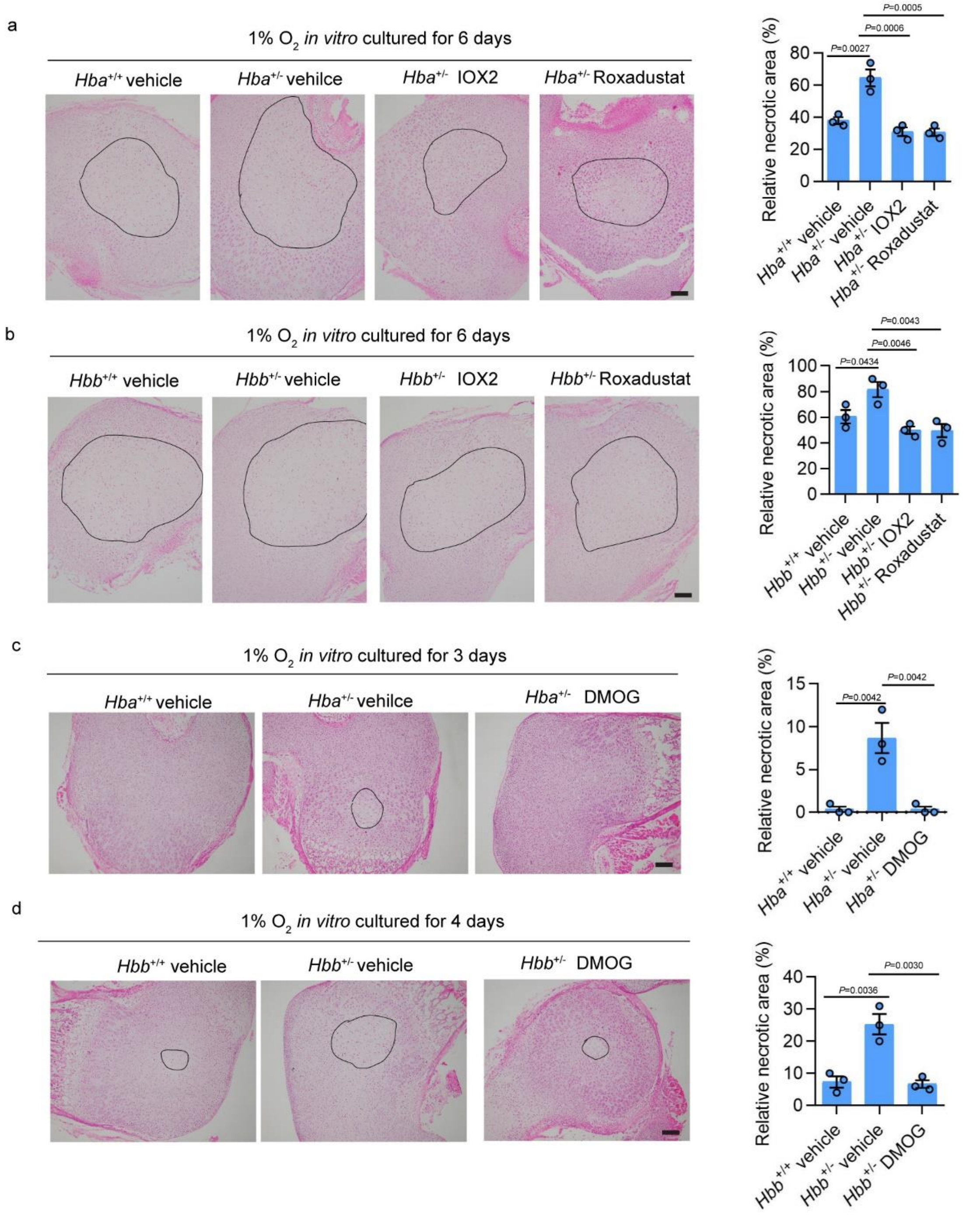

Supplementary Fig. 13 | Rescue of hemoglobin depletion-induced cell death by Hifs activation under hypoxia condition. a-d, Histology examination of proximal humeral cartilages of Ctrl (Hba$^{+/+}$ or *Hbb*$^{+/+}$), Hba$^{+/-}$ (**a**, **c**) or *Hbb*$^{+/-}$ (**b**, **d**) growth plates, which were treated by vehicle, IOX2 (50 μM), Roxadustat (50 μM) or DMOG (100 μM) in 1% O_2 for 3~6 days. Quantification of all biological replicates is provided on the right. Scale bars, 200 μm._n=3 biologically independent experiments. Error bars represent SEM. *P* values were calculated using one-way ANOVA tests. The exact *P*-values of comparison are presented in the figures, respectively.

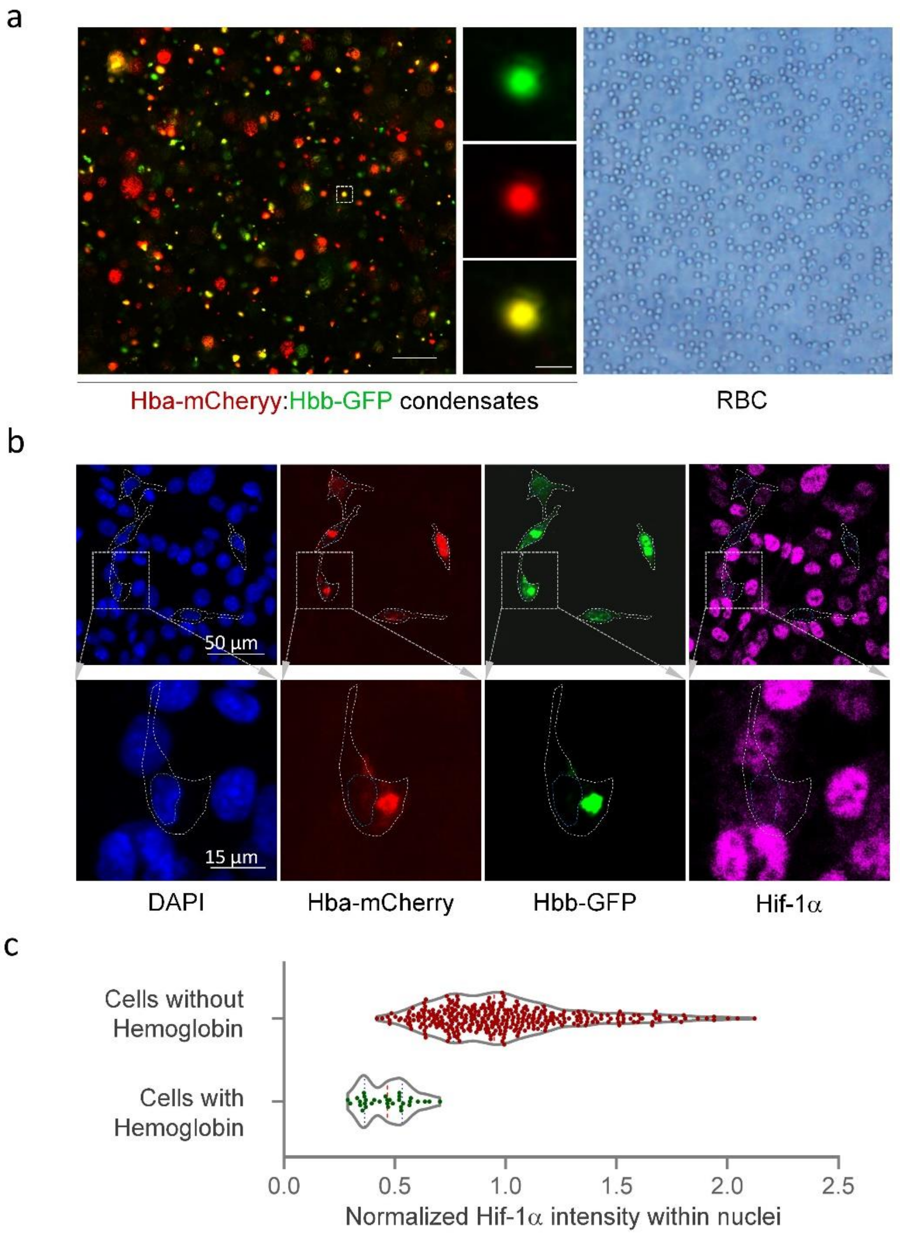

Supplementary Fig. 14 | Hypoxia rescue by hemoglobin body. a, Representative images of RBC (right panel) and hemoglobin condensates collected from 293T cells co-expressing Hba-mCherry and Hbb-EGFP (left panel for low magnification view, middle panel for zoomed view). Scale bar: 100 µm (left), 10 µm (middle). **b,** Representative images for Hif-1α staining in ATDC5 cells. Hif-1α in Deep pink, nuclei in blue, Hba-mCh in red, Hbb-EGFP in green. White dashed lines indicate outlines of foci positive cells, blue dashed lines indicate nulei of foci positive cells. Scale bars: 50 µm for the upper panel, 15 µm for the lower panel. **c,** Quantification of the nuclear Hif-1α intensities in ATDC5 cells. n =33 for foci positive cells, 341 for foci negative cells.
