# Supplementary material for: An extra-erythrocyte role of haemoglobin body in chondrocyte hypoxia adaption
Source: Nature. 2023 Oct 4;622(7984):834–41. doi: 10.1038/s41586-023-06611-6 (PMC10600011; doi:10.1038/s41586-023-06611-6)
Supplement: Supplementary file 2 — Reporting Summary [file 41586_2023_6611_MOESM2_ESM.pdf]

nature portfolio

Corresponding author(s): Qiang Sun; Feng Zhang

Last updated by author(s): Jul 25, 2023

Reporting Summary

Nature Portfolio wishes to improve the reproducibility of the work that we publish. This form provides structure for consistency and transparency in reporting. For further information on Nature Portfolio policies, see our Editorial Policies and the Editorial Policy Checklist.

Statistics

For all statistical analyses, confirm that the following items are present in the figure legend, table legend, main text, or Methods section.

n/a	Confirmed	
☐	☒	The exact sample size (*n*) for each experimental group/condition, given as a discrete number and unit of measurement
☐	☒	A statement on whether measurements were taken from distinct samples or whether the same sample was measured repeatedly
☐	☒	The statistical test(s) used AND whether they are one- or two-sided<br>*Only common tests should be described solely by name; describe more complex techniques in the Methods section.*
☒	☐	A description of all covariates tested
☒	☐	A description of any assumptions or corrections, such as tests of normality and adjustment for multiple comparisons
☐	☒	A full description of the statistical parameters including central tendency (e.g. means) or other basic estimates (e.g. regression coefficient) AND variation (e.g. standard deviation) or associated estimates of uncertainty (e.g. confidence intervals)
☐	☒	For null hypothesis testing, the test statistic (e.g. *F*, *t*, *r*) with confidence intervals, effect sizes, degrees of freedom and *P* value noted<br>*Give P values as exact values whenever suitable.*
☒	☐	For Bayesian analysis, information on the choice of priors and Markov chain Monte Carlo settings
☒	☐	For hierarchical and complex designs, identification of the appropriate level for tests and full reporting of outcomes
☒	☐	Estimates of effect sizes (e.g. Cohen's *d*, Pearson's *r*), indicating how they were calculated

Our web collection on statistics for biologists contains articles on many of the points above.

Software and code

Policy information about availability of computer code

Data collection	Western blot images were acquired and analyzed via the BioRad Image Lab system. Electron micrographs were captured by Gatan digital camera (832 SC1000, Gatan, Warrendale, PA, USA) and its application software (Gatan Digital Micrograph software v3.0) . All data files of LC-MS/MS were created using Bioworks Browser rev.3.1 (Thermo Electron, San Jose, CA.). The acquired MS/MS spectra were searched against the concatenated target/reverse Glycine max database using the SEQUEST search engine (Proteome Discoverer Software v2.3.0.523). Cells timelapse imaging were taken with the Ultraview Vox confocal system (Perkin Elmer, Volocity v6.3.0). Oxygenation-dissociation analyser and its application software (BLOODOX-2018 Analyser, Softron Biotechnology, Beijing, China)was used to determinate oxygen dissociation curve. Red blood cells (RBCs) counts and hemoglobin concentration (Hb) were performed by automatic blood analyzer (Sysmex Corporation, Japan, XP-100) and its application software (XT2000i1800i IPU). FRAP assay was conducted using the FRAP module of the Nikon confocal microscopy system (NIS Elements AR v4.50.00). Flow cytometry was performed with aflow cytometer (Coulter-XL, USA), EXPO32 ADC Software was used for data collection and analysis. The serial sections for correlative light electron microscopy were finally automatically acquired by a Helios Nanolab 600i dual-beam SEM (Scanning Electron Microscope, Thermo Fisher, USA) with an automated imaging software(Auto SEE 1.58) and Fluorescence images were collected by a confocal microscope (Zeiss LSM 980) and processed by accompanying software (ZEN v3.6095.01). Fluorescence quantitative PCR reaction was performed by Real time fluorescence quantitative PCR instrument (QTOWER3G, Jena Bioscience) and its application software (qPCRsoft v3.4).

Data analysis

Statistical analyses were performed using GraphPad Prism 8.0.1 or SPSS 18.0. Western blot quantification was performed using NIH Image J 1.8.0.112. Real-Time PCR results were analyzed by Microsoft Excel (2306 Build 16.0.16529.20164). The fluorescence intensity was quantified with Nikon confocal microscopy system (NIS Elements AR 4.50.00). RNA-seq data was analyzed and visualized using Fastp (version 0.14.1), Microsoft Excel (2306 Build 16.0.16529.20164), Hisat2 (version 2.2.0), circos (version 2.5.0), Stringtie (version 2.1.7), and edgeR (version 4.2.1).

For manuscripts utilizing custom algorithms or software that are central to the research but not yet described in published literature, software must be made available to editors and reviewers. We strongly encourage code deposition in a community repository (e.g. GitHub). See the Nature Portfolio guidelines for submitting code & software for further information.

Data

Policy information about availability of data

All manuscripts must include a data availability statement. This statement should provide the following information, where applicable:

- Accession codes, unique identifiers, or web links for publicly available datasets
- A description of any restrictions on data availability
- For clinical datasets or third party data, please ensure that the statement adheres to our policy

RNA-Seg data of the present study havebeen deposited in NCBI Gene ExpressionOmnibus (GEO) with accession codes GSE182640. Bioinformatics analysis of H3K4me3 modification region was performed at Cistrome Data Browser (http://cistrome.org/db/#/). Histone methylation data from work done by Ohba S et al (DOI: 10.1016/j.celrep.2015.06.013). Data of microdissection sample mass spectrometry, SDS-PAGE samplemass spectrometry, oligonucleotides and construct information have been deposited in supplementary tables.

Research involving human participants, their data, or biological material

Policy information about studies with human participants or human data. See also policy information about sex, gender (identity/presentation), and sexual orientation and race, ethnicity and racism.

Reporting on sex and gender

We report the patient whom cartilage was displayed in the manuscript. We did not do a analysis of sex-specific difference, due to sample size is rather limited.

Reporting on race, ethnicity, or other socially relevant groupings

We did not do a analysis of variable on groupings, due to sample size is rather limited. Human sample was not the main research in our manuscript. In addition,We did not find significant variable in animal experiments.

Population characteristics

Our study did not involve the population characteristics.

Recruitment

Human healthy articular cartilage specimens from patients with acute trauma were collected from the Department of Pathology of Xijing Hospital.

Ethics oversight

Human articular cartilage specimens were collected with an informed consent and approval of the project by the Research Ethics Board of the Xijing Hospital.

Note that full information on the approval of the study protocol must also be provided in the manuscript.

Field-specific reporting

Please select the one below that is the best fit for your research. If you are not sure, read the appropriate sections before making your selection.

[x] Life sciences   [ ] Behavioural & social sciences   [ ] Ecological, evolutionary & environmental sciences

For a reference copy of the document with all sections, see nature.com/documents/nr-reporting-summary-flat.pdf

Life sciences study design

All studies must disclose on these points even when the disclosure is negative.

Sample size

No statistical methods were used to predetermine sample size, which were chosen based on previous experiments and comparable studies in literature (PMID: 30651640, PMID: 32807933, PMID: 34163069). For key experiments, several test runs were performed to determine the suitable sample size. The sample sizes we chose are sufficient and suitable based on the following ingredients: 1. Effect size: the great difference in outcome measure between control and experimental groups (more than two-fold). 2. Variability: no considerable overlap in outcome measure between control and experimental groups. 3. Significance level : the statistical test Student's t-test and ANOVA with post-test used finds that the probability of the result is even lower than the chance of a type 1 error), indicating that the hypothesis is true. Sample size for each experiment is indicated in the legend.

Data exclusions

No exclusions other than caused by missing information have been made.

Replication

At least three biological replicates were performed for all in vivo experiments, most in vitro experiments were repeated at least three times. For non-quantitative data (micrographs, western blots and so on), findings were reproduced at least three times by replicating the

experiments and/or cross-validating with orthogonal approaches, and representative results are shown. Details of each exact number of replicates are provided in the figure legends.

Randomization

No statistical methods were used for randomization.
For in vitro experiments, cells were isolated from randomly chosen wild-type or knockout mice.
For in vivo experiments, mice were randomly allocated into experimental groups.

Blinding

Blinding was widely used in the study. Data collection and analysis, such as immunostaining, qRT-PCR, and Western blot were frequently performed by participants other than the experiment designer. During these data collection and analysis steps, all participants were routinely blinded to group allocation.

Reporting for specific materials, systems and methods

We require information from authors about some types of materials, experimental systems and methods used in many studies. Here, indicate whether each material, system or method listed is relevant to your study. If you are not sure if a list item applies to your research, read the appropriate section before selecting a response.

Materials & experimental systems

n/a	Involved in the study	
☐	☒	Antibodies
☐	☒	Eukaryotic cell lines
☒	☐	Palaeontology and archaeology
☐	☒	Animals and other organisms
☒	☐	Clinical data
☒	☐	Dual use research of concern
☒	☐	Plants

Methods

n/a	Involved in the study	
☒	☐	ChIP-seq
☐	☒	Flow cytometry
☒	☐	MRI-based neuroimaging

Antibodies

Antibodies used

Primary antibodies:
Epitope, Host Species , Concentration used, Manufacturer, Clone/Polyclonal, Catalog Number
Hif1a, Rabbit, Western Blotting 1:1000/Immunofluorescence 1:400, CST, D1S7W, 36169
Hbb, Rabbit, Western Blotting 1:1000/Immunofluorescence 1:400, Invitrogen, Polyclonal, PA5-60287
GFP, Rabbit, Western Blotting 1:1000, CST, Polyclonal, 2555
GFP, Rabbit, Electron Microscopy 1:100, Abcam, Polyclonal, ab290
Hba, Rabbit, Western Blotting 1:1000/Immunofluorescence 1:200, Invitrogen, SN70-09, MA5-32328
Hif2α/Epas1, Rabbit, Rabbit, Western Blotting 1:1000, Novus, polyconal, NB100-12
Klf1/Eklf, Rabbit, Western Blotting 1:1000, Abnova, ploycloncal, PAB5895
Klf1/Eklf, Mouse, ChIP-qPCR 5ug/ml, Active motif, 7B2, 61233
AMPKα1, Rabbit, Western Blotting 1:500, ABclonal, polyclonal, A1229
pAMPK,Rabbit, Western Blotting 1:500, ABclonal, ARC1547, AP1002
Caspase 3, Rabbit, Western Blotting 1:500/Immunofluorescence 1:200, ABclonal, ARC0133, A19654
Kdm5a, Rabbit, Western Blotting 1:500, ABclonal, ARC1120, A4755
Kdm5b, Rabbit, Western Blotting 1:500, ABclonal, polyclonal, A7772
H3K4me3, Rabbit, Chip-qPCR 5ug/ml, ABclonal, polyclonal, A2375
H3, Rabbit, chip-qPCR 5ug/ml, Proteintech, polyclonal, 17168-1-AP
Nonspecific IgG, rabbit, Chip-qPCR 5ug/ml, Proteintech, polyclonal, 30000-0-AP
α-Tubulin, rabbit, Western Blotting 1:3000, CST, p11H10, 2125

secondary antibodies
Epitope, Host Species , Concentration used, Manufacturer, Clone/Polyclonal, Catalog Number
Anti-Rabbit IgG (whole molecule)–Peroxidase antibody produced in goat, goat, 1:10000, Millipore, polyclonal, A0545
Anti-Mouse IgG (Fab specific) antibody produced in goat, goat, 1:10000, Millipore, polyclonal, M4155
Horseradish peroxidase enzyme (HRP)-coupled goat anti-rabbit secondary antibody, goat, 1:500, Abcam, polyclonal, ab7090

Validation

All antibodies were obtained from indicated commercial vendors with ensured quality. In addition, all the antibodies have been used in multiple experiments to detect intended proteins in control samples with expected molecular weight to validate their effectiveness in our study. Antibodies target for GFP were validated Specific affinity for EGFP in multiple literatures.
Primary antibodies:
Hif-1α, Reactivity: H, M, Mk. Application: WB IP,IF, F, ChIP, C&R. (https://www.cellsignal.cn/products/primary-antibodies/hif-1a-d1s7w-xp-rabbit-mab/36169?site-search-type=Products&N=4294956287&Ntt=36169&fromPage=plp&_requestid=5968605)
Hbb, Reactivity: H, highest antigen sequence identityto the following orthologs: Mouse-96%, Rat-96%. Application: IHC ; IICC/IF.(https://www.thermofisher.cn/cn/zh/antibody/product/HBB-Antibody-Polyclonal/PA5-60287)
GFP, Reactivity: species independent. Application: WB; IHC. (https://www.cellsignal.cn/products/primary-antibodies/gfp-antibody/2555?site-search-type=Products&N=4294956287&Ntt=2555&fromPage=plp&_requestid=5969550)
GFP, Reactivity: species independent. Application: WB; IHC; Electron Microscopy. (https://www.abcam.cn/products/primary-antibodies/gfp-antibody-ab290.html)

Hba Reactivity: H, M, Rat. Application: Western Blot (WB) ; IHC. (https://www.thermofisher.cn/cn/zh/antibody/product/Hemoglobin-alpha-Antibody-clone-SN70-09-Recombinant-Monoclonal/MA5-32328)
Hif-2a/Epas1, Reactivity: H, M, Rat, Fi, Ha, Pm, Rb, Re, Sh. Application: Chip; IHC; ELISA ; WB. (https://www.novusbio.com/products/hif-2-alpha-epas1-antibody_nb100-122)
Klf1/Eklf, Reactivity: M. Application: WB; .(https://www.abnova.com/products/products_detail.asp?catalog_id=PAB5895)
Klf1/Eklf, Reactivity: M. Application: chip-qPCR; (https://www.activemotif.com.cn/catalog/details/61233/eklf-antibody-mab-clone-7b2)
AMPKα1, Reactivity: H, M, Rat. Application: WB ; IF/ICC. (https://abclonal.com.cn/catalog/A1229)
p-AMPK, Reactivity: H, M. Application: WB. (https://abclonal.com.cn/catalog/AP1002)
Caspase3, Reactivity: H, M, Rat. Application: WB; IF/ICC. (https://abclonal.com.cn/catalog/A19654)
Kdm5a, Reactivity: H, M, Rat. Application: WB; IP; ChIP. (https://abclonal.com.cn/catalog/A4755)
Kdm5b, Reactivity: H, M. Application: WB; IF/ICC. (https://abclonal.com.cn/catalog/A7772)
H3K4me3, Reactivity: H, M, Rat. Application: WB; IF/ICC. (https://abclonal.com.cn/catalog/A2375)
H3, Rabbit, Reactivity: H, M, Rat. Application: FC, IF, IHC, IP, WB, ELISA. (https://www.ptgcn.com/products/Histone-H3-Antibody-17168-1-AP.htm)
Nonspecific IgG, Reactivity: H, M, Rat. Application: FC, IF, IHC, IP, WB, ELISA. (https://www.ptgcn.com/products/IgG-control-Antibody-30000-0-AP.htm)
α-Tubulin, Reactivity: H, M, Rat, Mk, Dm, Z, B, Pg. Application: IF, IHC, IP, WB. (https://www.cellsignal.cn/products/primary-antibodies/a-tubulin-11h10-rabbit-mab/2125?site-search-type=Products&N=4294956287&Ntt=2125&fromPage=plp&_requestid=5981232)
secondary antibodies
Anti-Rabbit IgG (whole molecule)–Peroxidase antibody produced in goat, Reactivity: R. Application: IF, IHC, WB. (https://www.sigmaaldrich.cn/CN/en/product/sigma/a0545)
Anti-Mouse IgG (Fab specific) antibody produced in goat, Reactivity: M. , Application: IF, IHC, WB. (https://www.sigmaaldrich.cn/CN/en/product/sigma/m4155)
Horseradish peroxidase enzyme (HRP)-coupled goat anti-rabbit secondary antibody, Reactivity: R. Application: ICC/IF, IHC-P, WB, ELISA, Immunomicroscopy, Dot blot, IHC-Fr. (https://www.abcam.cn/products/secondary-antibodies/goat-rabbit-igg-hl-hrp-preadsorbed-ab7090.html)

Eukaryotic cell lines

Policy information about cell lines and Sex and Gender in Research

Cell line source(s)	293T, HepG2, HCT116, PLC/PR/F5, PC12 cell lines were purchased from Procell Life Science&Technology Co.,Ltd. ATDC5 cell line was purchased from NingboMingZhoubioCO.,Ltd, which was derived from ECACC.
Authentication	All cell lines were authenticated by STR profiling.
Mycoplasma contamination	All cell lines were mycoplasma-free after PCR verification.
Commonly misidentified lines (See ICLAC register)	There is no commonly misidentified cell line were used in the study.

Animals and other research organisms

Policy information about studies involving animals; ARRIVE guidelines recommended for reporting animal research, and Sex and Gender in Research

Laboratory animals

Mice heterozygous for the alpha-globin null allele (Hba+/-, both of the adult hemoglobin genes, alpha 1 and alpha 2, and the region between them were deleted and replaced with a neomycin resistance cassette by homologous recombination) or for the beta-globin null allele (Hbb+/-, a genomic fragment encompassing all of Hbb-b1 and a 5' portion of Hbb-b2 was replaced with a neo cassette inserted by homologous recombination.) were produced by crossing mice of Hbatm1Paz Hbbtm1Tow Tg (HBA-HBBs) 41Paz/J (Jackson Labs, No:003342)46 with wildtype C57BL/6J mice. The Hbatm1Paz Hbbtm1Tow Tg (HBA-HBBs) 41Paz/J mice are called sickle cell mice (Berkeley model), which are homozygous for the both alpha-globin and beta-globin null allele and carrying the human sickle transgene (Hba0/0 Hbb0/0Tg (Hu-miniLCRα1GγAγδβS). Mice homozygous for Hba or Hbb knock-out mutation die in utero for severe anemia. Hif-1aF/F (No.007561)47, Col2a1-CreERT2 (No.006774)48, Prx1-Cre (No.005584)49 mice were from Jackson Labs. Hif-2αF/F (No.NM-CKO-200163) mice were from Shanghai Model Organisms Center, Inc. To specifically knockout Hif-1α or/and Hif-2α gene in chondrocytes, Hif-1αF/F or/and Hif-2αF/F mice were bred to Col2a1-CreERT2 mice. To conditionally delete Hbb in mesenchymal cells or chondrocytes, HbbF/F mice were bred to Prx1-Cre or Col2a1-CreERT2 mice. To investigate the role of Klf1 in chondrocytes, we generated conditional Klf1-floxed mice with C57BL/6J-background by homologous recombination. A targeting vector was designed to replace the 2nd exon of Klf1 (Sup. Fig. 3a). To conditionally delete Klf1 in chondrocytes, Klf1F/F mice were bred to Col2a1-CreERT2.

Studies were conducted on E14.5 to 12 month old mus musculus of the C57BL/6 Strain.

Mice were housed in a room on a 12:12 light:dark cycle, a 72+/-2 F temperature setting and humidity of 30-70%.

Wild animals	There is no wild animal in our study.
Reporting on sex	Sex-based analyses were not performed, due to no obvious sex and gender difference was observed in our study.

Field-collected samples: There is no field collected samples were used in the study.

Ethics oversight: Animal studies were approved by the Institutional Animal Care and Use Committee at the Fourth Military Medical University.

Note that full information on the approval of the study protocol must also be provided in the manuscript.

Flow Cytometry

Plots

Confirm that:

- [x] The axis labels state the marker and fluorochrome used (e.g. CD4-FITC).
- [x] The axis scales are clearly visible. Include numbers along axes only for bottom left plot of group (a 'group' is an analysis of identical markers).
- [x] All plots are contour plots with outliers or pseudocolor plots.
- [x] A numerical value for number of cells or percentage (with statistics) is provided.

Methodology

Sample preparation: Analysis of total ROS: Primary chondrocytes were used to guantify total ROS accumulation with a ROS assav kit (Cayman Chemicals) according to the manufacturer's instructions. After a brief trypsinization with Trypsin-EDTA (0.05%) at 37℃,cells were incubated in dark with 5mM dihydroethidium (DHE) probe for 30 minutes at 37℃.Thereafter,cells were washed twice with ROS staining buffer.
Analysis of mitochondrial ROS : According to the manufacturer's instructions, the mitochondrial ROS accumulation was performed with a MitoSOXassay kit (Invitrogen). Briefly, primary chondrocytes isolated and cultured were incubated with 5mM MitoSOX probe for 20 minutes at 37℃ in dark.Aftera brief trypsinization with Trypsin-EDTA (0.05%) at 37℃,cells were washed twice with FACS buffer (1x DPBS +5% FBS).

Instrument: Flow cytometry was performed with a flow cytometer(Coulter-XL, USA).

Software: EXPO32 ADC Software was used for analysis.

Cell population abundance: Flow sorting was not performed.

Gating strategy: Cells were gated by FSC-A x SSC-A to exclude debris and then by FSC-H x FSC-W following SSC-H x SSC-W to exclude cell doublets.
1. Set a gate based on the negative control (with a confidence interval of about 2% for the negative control) 2. When the fluorescence signal of the test sample is greater than the negative control, it is defined as positive.

- [x] Tick this box to confirm that a figure exemplifying the gating strategy is provided in the Supplementary Information.
