# Supplementary material for: An extra-erythrocyte role of haemoglobin body in chondrocyte hypoxia adaption
Source: Nature. 2023 Oct 4;622(7984):834–41. doi: 10.1038/s41586-023-06611-6 (PMC10600011; doi:10.1038/s41586-023-06611-6)
Supplement: Supplementary file 6 — Oligonucleotides. [file 41586_2023_6611_MOESM6_ESM.docx]

**Table S3 Oligonucleotides**

| Target | Forward primer | Reverse primer |
| --- | --- | --- |
| *Primers used for genotyping PCR* | | |
| *Hbb*-flox | TGCATCTGCAGATCCCAAAAA | GGAGGAGTGTACAAGGAGTTCAATAA |
| *Col2a1*-cre^ert2^ | CACTGCGGGCTCTACTTCAT | ACCAGCAGCACTTTTGGAAG |
| *Hba+/+* | ATAGATGGGTAGCCATTTAGATTCC | CCGGGTTATAATTACCTCAGGTC |
| *Hba+/-* | ATAGATGGGTAGCCATTTAGATTCC | CCGGGTTATAATTACCTCAGGTC |
| *Hbb+/+* | ACCCACAGCCAAACACTAGG | GAAGGGAAGAAGCAACATGC |
| *Hbb+/-* | AGAATAGCCAGGGGAAGGAA | CTGTCCATCTGCACGAGACT |
| *Tg-HBA-HBB* | TCAAGGAGGCAAATGGAAAC | AGGCACCTAGCACCTTCTTG |
| *Hif-1α-flox* | TGCATGTGTATGGGTGTTTTG | GAAAACTGTCTGTAACTTCATTTCC |
| *Prx1* | TCTCTGGCTCTGATGTTGGCA | CGCATAACCAGTGAAACAGC |
| *Hif-2α-flox* | TTGCAAATGGAGGCGTGTTG | CTCTGCTTTTGCGCATTGGT |
| *Klf1-flox* | AGGGGTCTGAGATCAAGGTGA | CGGTTCCCCTAACCCCTTTC |
| *RT-qPCR for mouse samples* | | |
| *Gaphd* | TGAAGCAGGCATCTGAGGG | CGAAGGTGGAAGAGTGGGAG |
| *Hif-1a* | CTATGGAGGCCAGAAGAGGGTAT | CCCACATCAGGTGGCTCATAA |
| *Hif-2a* | CTGAGGAAGGAGAAATCCCGT | TGTGTCCGAAGGAAGCTGATG |
| *Vegfa* | CTTGTTCAGAGCGGAGAAAGC | ACATCTGCAAGTACGTTCGTT |
| *EPO* | CATCTGCGACAGTCGAGTTCTG | CACAACCCATCGTGACATTTTC |
| *EPOR* | AAACTCAGGGTGCCCCTCTGGCCT | GATGCGGTGATAGCGAGGAGAACC |
| *alpha-globin* | TTGGCTAGCCACCACCCT | CCAAGAGGTACAGGTGCA |
| *beta-maj-globin* | TTAAGGCTCCTGGGCAATAT | TGCCAACAACTGACAGATGC |
| *epslo-globin* | GTTTTGGCTAGTCACTTCGG | CAAGGAACAGCTCAGTATTC |
| *zeta-globin* | GAAGCCTGGGACAAGTTCAT | GGGTTCAATAAAGGGGAGGA |
| *beta-h1-globin* | TTGCCAAGGAATTCACCCCA | CTCAATGCAGTCCCCATGGA |
| *Klf1* | CAGCTGAGACTGTCTTACCC | AATCCTGCGTCTCCTCAGAC |
| *Runx1* | GCCTCTCTGCAGAACTTTCC | GACGGCAGAGTAGGGAACTG |
| *Gata-1* | AGGCCCTGGAAGACCAGGAAG | AGAAAGGACTGGGAAAGTCAGC |
| *εy-globin-f2* | ACCCTCATCAATGGCCTGTGGA | CATGGGCTTTGACCCTTGGG |
| *βh1-globin* | ATCATGGGAAACCCCCGGA | GGGTGAATTCCTTGGCAAAATGAGT |
| *Col2a1* | CCAGGGCTCCAATGATGTAG | GCGGGAGGTCTTCTGTGATC |
| *Sox9* | CCAGCAAGAACAAGCCACAC | TCTCGTTCAGCAGCCTCCAG |
| *Runx2* | TGACATCCCCATCCATCCAC | AGAAGTCAGAGGTGGCAGTG |
| *Kdm5a* | CACAGACCCGCTGAGTTTTAT | CTTCACAGGCAAATGGAGGTT |
| *Kdm5b* | AAGCCAAGCTCTGTTCAGCAA | GAAGGCAATCGTTCTTCTCACT |
| *Bap1* | CTCCTGGTGGAAGATTTCGGT | GAGTGGCACAAGAGTTGGGAA |
|  |  |  |
| *ChIP-qPCR in mouse samples* | | |
| α MRE (HS26) (311 bp), | ACCCATCTGGAACCTATGAG | GAAAGTCTTCCCAACTGCAG |
| α1 promoter region (243 bp), | CTTCCCAAACTGCCATCACT | TCTTGGACCTGCAGCAAGTA |
| HS1 | CAGATCCTCAAACACTCTCCCATAA | TGCCTTCTTTGTCCCATCATT |
| HS2 | GGGTGTGTGGCCAGATGTTT | CACCTTCCCTGTGGACTTCCT |
| HS3 | CTAGGGACTGAGAGAGGCTGCTT | ATGGGACCTCTGATAGACACATCTT |
| βmaj promoter regions (217 bp), | GACAAACATTATTCAGAGGGAGTA | AAGCAAATGTGAGGAGCAACTGAT |
| *Klf1* | GTGGGACCCGGATCTTTTCC | AAAGGCCCAGTCACCAACC |
| *Bap1* | CGTCGTGTGATGATGACGTTG | TATTCATCTTCCCGCGGAGC |
|  |  |  |
| *siRNA for mouse Klf1* | | |
| Klf1-mus-149 | CAUCACGUGAGUCUGAAAUTT | AUUUCAGACUCACGUGAUGTT |
| Klf1-mus-17 | GUCUUACCCUCCAUCAGUATT | UACUGAUGGAGGGUAAGACTT |
| Kdm5a-mus-1062 | GCCUGAUUUAGAUCUUAAATT | UUUAAGAUCUAAAUCAGGCTT |
| Kdm5a-mus-3981 | GCGCAUAGAAGAAGUGAAATT | UUUCACUUCUUCUAUGCGCTT |
| Kdm5b-mus-2432 | GGGCCUUAAAUGUGAAUGATT | UCAUUCACAUUUAAGGCCCTT |
| Kdm5b-mus-1532 | GCUGGAAUUUGAACAACAUTT | AUGUUGUUCAAAUUCCAGCTT |

| *Primers used for recombination ratio* | | |
| --- | --- | --- |
| *Hbb*-flox | CTTGGACCCAGCGGTACTTT | ATGCAGCTTGTCACAGTGGA |
| *Hba-/-* | TTCCCCACCACCAAGACCTA | AAGTTGACGGGATCCACACG |
| *Hbb-/-* | CTTGGACCCAGCGGTACTTT | ATGCAGCTTGTCACAGTGGA |
| *Hif-1α-flox* | GATCTCGGCGAAGCAAAGAGT | CACATTGTGGGGAAGTGGCAA |
| *Hif-2α-flox* | GCTCAGAGCTGAGGAAGGAG | ATGGAGGCTTTGTCCAGGTG |
| *Klf1-flox* | GAGGAGACGCAGGATTTGGG | CGGAAGGGTCCTCCGATTTC |
| *Hprt-internal reference* | TTGACCCGACTGATGGTTCC | CCAGCAAGAAGTGTCACCGT |
| *β2-Microglobulin* | TCATTAGGGAGGAGCCAATG | ATCCCCTTTCGTTTTTGCTT |
