# Supplementary material for: An extra-erythrocyte role of haemoglobin body in chondrocyte hypoxia adaption
Source: Nature. 2023 Oct 4;622(7984):834–41. doi: 10.1038/s41586-023-06611-6 (PMC10600011; doi:10.1038/s41586-023-06611-6)
Supplement: Supplementary file 7 — Construct information. [file 41586_2023_6611_MOESM7_ESM.docx]

**Table S4 construct information**

| No. | **Plasmid Name** | **Vector** | **Site** | **DNA** | **Primer** | **Primer Sequence (5'→3')** |
| --- | --- | --- | --- | --- | --- | --- |
| 1 | pQCXIP-*Hbb*-EGFP | pQCXIP-EGFP | XhoI | Hbb | Hbb-PQC-F | GCGGCCGCACCGGGATCATGGTGCATCTGACTCCTGAGG |
|  |  |  | Age I |  | Hbb-EGFP-R | CCTTGCTCACCATGGTGGCGATGTGATACTTGTGGGCCAGGGC |
| 2 | pQCXIP-*Hba1*-mCh | pQCXIP-mCherry | XhoI | Hba1 | Hba-PQC-F | TGCAGGAATTGATCCGCGGCCGCACCGGGATCATGGTGCTCTCTGGGGAAGACAA |
|  |  |  | Age I |  | Hba-mCh-R | CTCCTCGCCCTTGCTCACCATGACCGGTGAACGGTACTTGGAGGTCAGCAC |
| 3 | pQCXIP-*Hbb*(∆N)-EGFP | pQCXIP-EGFP | XhoI | ∆N-1 | hbb-D1-F | AGCTCAAGCTTCGAATTCGCCACATGTCTGCCGTTACTGCCCTGTGG |
|  |  |  | Age I | ∆N-2 | Hbb-EGFP-R | CCTTGCTCACCATGGTGGCGATGTGATACTTGTGGGCCAGGGC |
| 4 | pQCXIP-*Hbb*(∆C)-EGFP | pQCXIP-EGFP | XhoI | ∆C-1 | PQC-F | AGCTCGTTTAGTGAACCGTCAGATCG |
|  |  |  | Age I | ∆C-2 | hbb-D4-R | GCCCTTGCTCACCATGGTGGCGACAGCCACACCAGCCACCAC |
| 5 | pQCXIP-*Hbb*(∆N&∆C)-EGFP | pQCXIP-EGFP | XhoI | ∆N&∆C-1 | hbb-D1-F | AGCTCAAGCTTCGAATTCGCCACATGTCTGCCGTTACTGCCCTGTGG |
|  |  |  | Age I | ∆N&∆C-2 | hbb-D4-R | GCCCTTGCTCACCATGGTGGCGACAGCCACACCAGCCACCAC |
| 6 | pQCXIP-*Hbb*(A139P)-EGFP | pQCXIP-EGFP | XhoI | A139P-1 | PQC-F | AGCTCGTTTAGTGAACCGTCAGATCG |
|  |  |  |  |  | Hbb-B-R | GCCAGGGCATTTGGCACACCAGCCACCACTTTC |
|  |  |  | Age I | A139P-2 | Hbb-B-F | CCAAATGCCCTGGCCCACAAGTATC |
|  |  |  |  |  | Hbb-EGFP-R | CCTTGCTCACCATGGTGGCGATGTGATACTTGTGGGCCAGGGC |
| 7 | pQCXIP-*Hbb* | pQCXIP-EGFP | XhoI | HBB | PQC-F | AGCTCGTTTAGTGAACCGTCAGATCG |
|  |  |  | Mfe I |  | hbb-T-PQC-R | GGGGCGGAATTAACAACAACTTAGTGATACTTGTGGGCCAGGGCATTAGCC |
| 8 | pQCXIP-*Hbb*-EGFP  (Optimized codon for *E. coli*) | PET-24(+) | NdeI | Hbb-EGFP-O | HBB-PET-F | TTTGTTAGCAGCCGGATCTCAATGATATTTATGTGCCAGGGCATTTG |
|  |  |  | XhoI |  | HBB-PET-R | AGAAGGAGATATACATATGCACCACCACCACCACCACATGGTTCATCTGACCCCGGAAG |
